# Riding the Wave: The SINE-Specific V Highly-Conserved Domain Spread into Mammalian Genomes Exploiting the Replication Burst of the MER6 DNA Transposon

**DOI:** 10.3390/ijms20225607

**Published:** 2019-11-09

**Authors:** Andrea Luchetti, Mariana Lomiento, Barbara Mantovani

**Affiliations:** 1 Department of Biological, Geological and Environmental Sciences, University of Bologna, 40126 Bologna, Italy; barbara.mantovani@unibo.it; 2 Sant’Orsola Malpighi Hospital, University of Bologna, 40138 Bologna Italy; mariana.lomiento@aosp.bo.it

**Keywords:** highly-conserved domains, Mammalia, Tc1/Mariner transposon, short interspersed elements (SINEs), miniature inverted-repeat transposable element (MITE)

## Abstract

Transposable elements are widely distributed within genomes where they may significantly impact their evolution and cell functions. Short interspersed elements (SINEs) are non-autonomous, fast-evolving elements, but some of them carry a highly conserved domain (HCD), whose sequence remained substantially unchanged throughout the metazoan evolution. SINEs carrying the HCD called V are absent in amniote genomes, but V-like sequences were found within the miniature inverted-repeat transposable element (MITE) MER6 in *Homo sapiens*. In the present work, the genomic distribution and evolution of MER6 are investigated, in order to reconstruct the origin of human V domain and to envisage its possible functional role. The analysis of 85 tetrapod genomes revealed that MER6 and its variant MER6A are found in primates, while only the MER6A variant was found in bats and eulipotyphlans. These MITEs appeared no longer active, in line with literature data on mammalian DNA transposons. Moreover, they appeared to have originated from a Mariner element found in turtles and from a V-SINE from bony fishes. MER6 insertions were found within genes and conserved in mRNAs: in line with previous hypothesis on functional role of HCDs, the MER6 V domain may be important for cell function also in mammals.

## 1. Introduction

Transposable elements (TEs) are DNA sequences able to replicate and insert within genomes [1,2]. Since their first discovery [3], TEs are known to impact host genome evolution and cell functions in several ways; for example, they can modify gene regulatory networks, mediate genomic rearrangement or even be exapted as new genes or gene components [2]. They are distributed across all living being genomes and can be classified on the basis of their replication mechanisms and sequence structures [1]. Class I TEs replicate via an RNA intermediate, through copy-and-paste mechanisms, while class II TEs proliferate through a DNA intermediate, using different molecular strategies. Both classes include autonomous and non-autonomous elements: the former are able to code for the protein(s) necessary for their replication/integration, while the latter have to exploit the enzymatic machinery of an autonomous partner element. 

Short interspersed elements (SINEs) and miniature inverted-repeat transposable elements (MITEs), which belong to class I and class II, respectively, are the most successful non-autonomous elements [4,5]. SINEs are widespread among vertebrates and they were found well-represented in cartilaginous fishes, the coelacanth and mammals [6]. They are very short sequences (200–600 bp) composed by three “modules”: an RNA-related head, which may originate from tRNA, 7SL, or 5S rDNA genes, a tail homologous to that of long interspersed repeats (LINEs), which allows to exploit the LINE reverse transcriptase, and a body linking the head and the tail [4]. The origin of the body is unknown in the majority of SINEs, although a LINE-derived origin has been proposed [7]. Surprisingly, it has been observed that some SINEs, having different origins and with different taxonomic distributions, may share a highly-conserved domain (HCD) [4,8]. Nine HCDs have been discovered so far in vertebrates, echinoderms, arthropods, molluscs, annelids and cnidarians [8,9,10,11,12,13,14,15,16]. The discovery of HCDs in fast evolving sequences like SINEs, raised the question of why they are highly conserved. Many hypotheses have been put forward in the last two decades, including the possibility to mediate the SINE-LINE tail exchange [17], the module exchange [8], or even conferring an advantage to the host genome [18]. In this view, it has to be considered that some HCD-SINE underwent exaptation, being domesticated as gene enhancers [19,20,21,22]. Moreover, an implication for some HCDs, such as CORE, Deu and V domains, as regulatory elements has been suggested based on mRNAs and miRNA genes analyses in fishes [23,24]. 

SINEs carrying the ~120 bp long V HCD are among the most widespread among metazoans, but they apparently lack in amniote genomes; on the other hand, the V domain has been found within a DNA transposon, called MER6, in the human genome [10,16]. Ogiwara and co-workers [10] concluded that V-homologous sequences could be either a genomic debris derived from a V-SINE inserted into the DNA element, or could have been conserved for some, yet uncharacterized, functional advantage for the host genome. The DNA transposon MER6 has been classified in Repbase Update, the database of repeated DNAs [25], and in previous literature [26] as a Tc1/Mariner DNA element. MER6 does not show any protein coding potential, as also suggested by its short length (865 bp); it shows two terminal inverted repeats (TIR) which are 24 bp long and insertions are surrounded by TA target site duplication [26]. Overall, these features help to identify MER6 as a MITE [27]. Moreover, MER6 has a homologous element, called MER6A: the consensus sequences of the two MITEs diverge by 0.3%, and the only structural difference is a 261 bp long internal deletion in the MER6A repeat.

As both MER6 and MER6A (henceforth collectively referred to as simply MER6) show the conserved V domain, it is interesting to investigate about their origin, including that of the V HCD, to highlight their evolutionary dynamics. For this purpose, a genome-wide analysis was undertaken on representative genomes of amniote (mammals, reptiles and birds) and non-amniote vertebrates (amphibians). The actual taxonomic distribution of MER6 as well as the parental TEs that gave origin to MER6 are reported here. Moreover, similarly to some HCD-carrying SINEs, MER6 elements were also found embedded in messenger RNAs (mRNAs).

## 2. Results

### 2.1. MER6/MER6A Taxonomic Distribution

**Figure 1:**
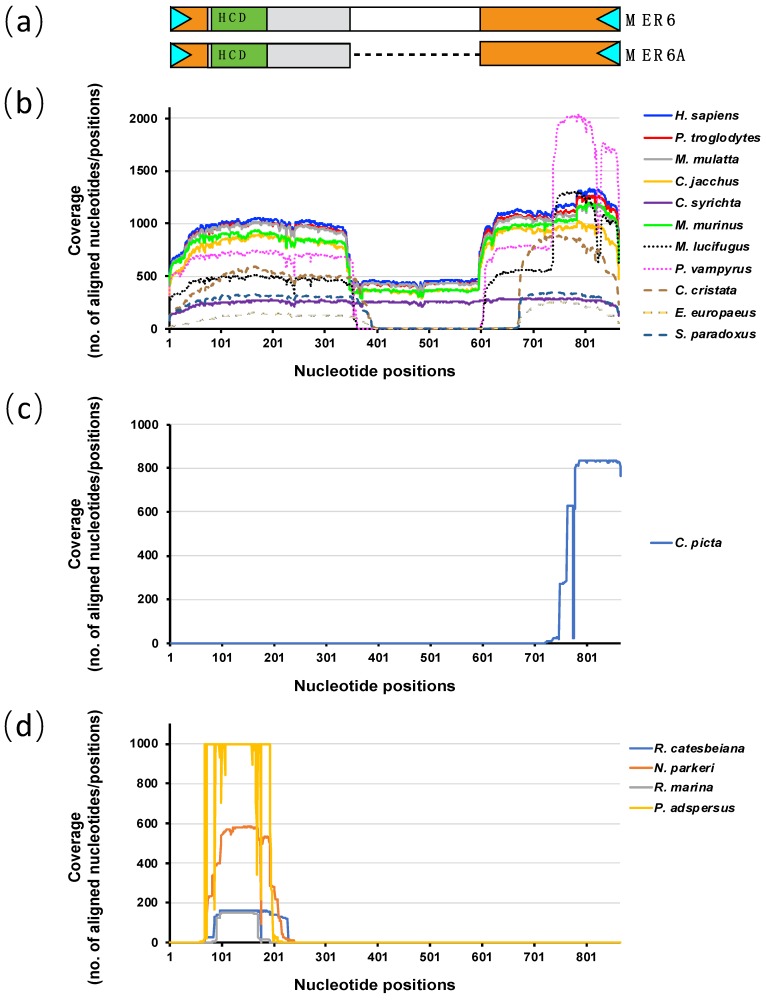
MER6 elements coverage from BLAST significant alignments. The schematic structure of MER6 elements is reported in (**a**) (cyan triangles: terminal inverted repeats; orange boxes: regions homologous to Mariner-2_CPB; grey boxes: regions homologous to Ac1 SINEs; green boxes: V highly conserved domains (HCD)). Sequence coverage from BLAST alignments with mammalian genomes (**b**), reptile genomes (**c**) and amphibian genomes (**d**).

The presence of MER6 elements was checked by BLAST on 85 representative tetrapod genomes: 77 amniotes (43 mammals, 21 birds and 13 reptiles) and eight amphibians (Figure 1).

In mammals, from 385 to 2923 significant BLAST alignments, covering the entire length of MER6 and MER6A, were found in primates (*Homo sapiens*, *Pan troglodytes*, *Macaca mulatta*, *Callithrix jacchus*, *Carlito syrichta* and *Microcebus murinus*), bats (*Pteropus vampyrus* and *Myotis lucifugus*) and eulipotyphlans (*Erinaceus europaeus*, *Condylura cristata* and *Solenodon paradoxus*) (Figure 1b). MER6 sequences coverage resulting from BLAST alignments showed a drop corresponding to the 261 bp long internal deletion in the MER6A element; in particular, while searching in bats and eulipotyphlans the coverage became zero. Moreover, it seems that in eulipotyphlans the length of the drop region was slightly larger than in bats (Figure 1b).

In the remaining mammalian genomes, no hits were recorded. The same result was obtained when searching in bird genomes, while small fragments of 70–120 bp, located at the 3’ end of MER6 elements, significantly matched with sequences in some reptilian genomes: in particular, from 1 to 6 BLAST alignments were retrieved in *Gavialis gangeticus*, *Alligator sinensis*, *Crocodylus porosus* and *Pelodiscus sinensis*, while 833 BLAST alignments were found in the genome of the turtle *Chrysemys picta* (Figure 1c).

In amphibian genomes, significant alignments were recorded against the genome of the anuran (frogs and toads) species *Rana catesbeiana*, *Nanorana parkeri*, *Pyxicephalus adspersus* and *Rhinella marina*. These were restricted to a 60–170 bp fragment located between base 60 and 240 of MER6 repeats and corresponding to the V HCD region (Figure 1d). Genomes inspection revealed that the significant alignments corresponded, in fact, to V-SINEs related to the *Racla* element isolated from the green frog *Rana clamitans* [16]: the Jukes–Cantor divergence from *Racla* ranged from 2.8% (*Racla* vs. *Rana catesbeiana*) to 24.4% (*Racla* vs. *Pyxicephalus adspersus*) (Supplementary Figure S1; Supplementary Data S1). Moreover, one out of two caecilian species, *Rhinatrema bivittatum*, exhibited 11 significant BLAST alignments spanning from base 88 to 372 of MER6 consensus sequences. However, a close inspection of genes surrounding the positive hits in the *R. bivittatum* contigs revealed a closer similarity with fish genomes (not shown). Since these unexpected results would deserve a deeper analysis, which go beyond the scope of the present work, these alignments were not further considered.

### 2.2. MER6 and MER6A Copy Number and Nucleotide Variability

In order to obtain a more complete picture of MER6 element occurrence in mammalian genomes, primate, chiropteran and eulipotyphlan genomes were analysed through RepeatMasker and fragments corresponding to MER6 elements isolated and aligned. Primates were found to contain both MER6 and MER6A, with copy numbers ranging from 364 to 428 and from 482 to 648, respectively; therefore, they cover up to the 0.011% of the genome (Table 1). In bats and eulipotyphlans only MER6A was found, with the number of insertions ranging from 136 to 873. Overall, the two bat genomes appeared to host more repeats than those of insectivores, with the megabat *P. vampyrus* genome having the highest MER6A coverage (0.023%) (Table 1). The Jukes–Cantor divergence among repeats within each genome ranged from 32.3% (MER6A in *P. vampyrus*) to 65.6% (MER6A in *E. europaeus*) (Supplementary Table S1).

Consensus sequences obtained from isolated MER6 copies do not result in large differences among assayed genomes, with the exception of eulipotyphlan ones: as expected from the BLAST alignments analyses (Figure 1b), MER6A from *C. cristata*, *E. europaeus* and *S. paradoxus* showed a larger deletion than primates and chiropteran MER6A consensus (Supplementary Figure S2). Overall, MER6 elements exhibited a Jukes–Cantor divergence from the consensus ranging from 19.2% to 22.9%; MER6A elements showed approximately a comparable range of variability in primate and bat genomes, but they were more divergent in eulipotyphlan genomes (27.6%–34.9%; Table 1). The distribution of divergences from the consensus sequence clearly suggested that the two MITEs have been recently inactive in all assayed genomes, as the less divergent copies from the consensus showed more than 12% Jukes–Cantor distance from the consensus (Supplementary Figure S3).

**Figure 2:**
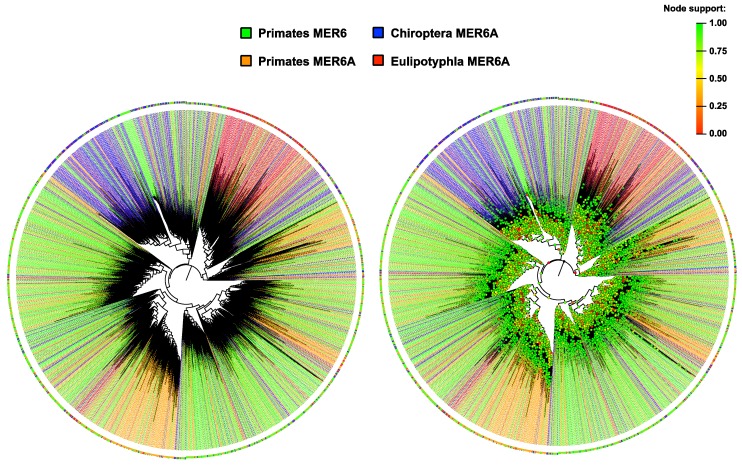
Maximum likelihood phylogenetic analysis on 2438 MER6 and the 6150 MER6A copies isolated from primates, chiropterans and eulipotyphlans. In the left panel, the maximum likelihood tree calculated using the Jukes–Cantor+CAT model without node supports for improving the graphical clarity. In the right panel, the same phylogenetic tree with node support represented by coloured dots as per the upper-right legend.

The 2438 MER6 and the 6,150 MER6A copies, for a total of 8588 repeat sequences, were aligned and used to build a maximum likelihood phylogenetic tree with two different substitution models (Figure 2; Supplementary Figure S4). The two phylogenetic trees were fully congruent; the majority of nodes were supported but failed to identify a meaningful clustering pattern. MER6 and MER6A sequences do not result separated into repeat-specific clades, nor are they distributed in clades representing a particular taxonomic pattern (species or order; Figure 2; Supplementary Figure S4).

### 2.3. MER6 Occurrence within Genic Regions

**Figure 3:**
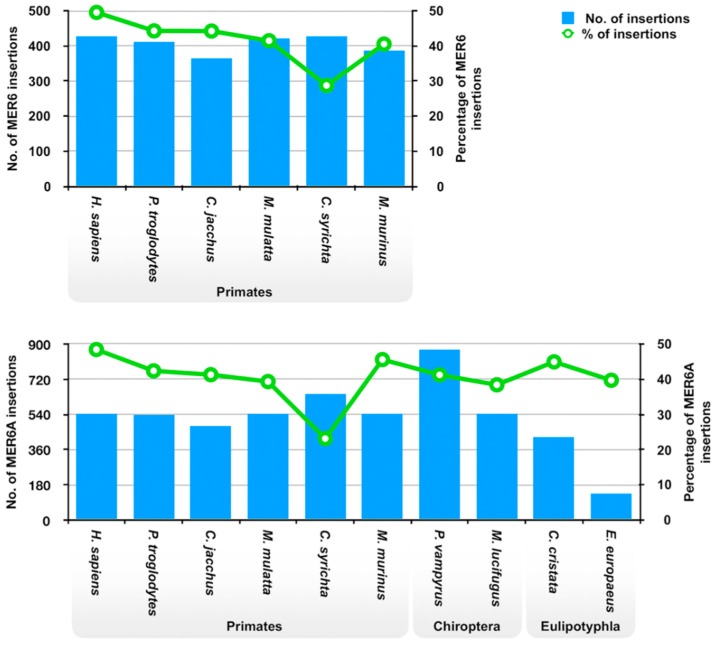
Genomic distribution of MER6 elements in assayed genomes. Number of insertions (blue bars) and percentage of insertions (green line) occurring within genic regions (annotated genes ± 5000 bp flanking regions).

The genomic distribution of MER6 and MER6A elements was analysed by checking the frequency of insertions within genic regions. On average, the two MITEs showed the 41.5% and 40.5% of insertions within genes plus flanking regions, respectively, although this percentage may vary among genomes (Figure 3). In particular, the fraction of MER6 and MER6A elements inserted within gene flanking regions resulted less than the 5% of the total number of insertions (Supplementary Figure S5). Overall, the genomic distribution of both MER6 and MER6A appeared dependent from the species, as it resulted significantly different among species (Chi-square test for independence, *p* < 0.001). When considering all possible pairwise comparison, it emerged that *C. syrichta* consistently showed a significantly lower percentage of both MER6 and MER6A insertions within genes plus flanking regions (post-hoc pairwise test with Bonferroni correction, *p* < 0.01).

The occurrence of MER6 and MER6A elements within transcriptomes revealed that they are mostly present in mRNAs; at variance of all other species, in *H. sapiens* the two MITEs are also equally represented as long non-coding RNAs (lncRNAs) (Table 2). In two mRNA from *P. troglodytes* and *C. jacchus* MER6 BLAST significant alignments occurred within the protein coding regions (Supplementary Figure S6), while the remaining BLAST alignments occurred within the 5’ or 3’ untranslated regions (UTRs) (Table 2).

### 2.4. MER6 Composite Structure

Using MER6 as a query to BLAST to search in the Repbase database, a significant alignment against an autonomous Tc1/Mariner element from the painted turtle *Chrysemys picta* (Mariner-2_CPB) was retrieved. In particular, the extent of similarity regions spanned the 5’ end (from 56 bp to 57 bp long) and the 3’ end (from 101 bp to 104 bp long) (Supplementary Figure S7), with a sequence divergence ranging from 31.6% (5’ end) to 17.6% (3’ end). The autonomous Tc1/Mariner element does not show homologous copies in non-turtle genomes. When the Mariner-2_CPB sequence was used to probe the Genbank nr/nt database, positive alignments were found for the central part of the element containing the open reading frame coding for the transposase protein. If the search is limited only to mammalian sequences, the extent of BLAST alignments is even more reduced (Supplementary Figure S8).

Further significant BLAST alignments were found against the Ac1 SINE from *Takifugu rubipes* [10]: the extent of the similarity region spanned from the whole body (which include the V HCD) to the tail of the fugu’s SINE, thus excluding the tRNA-related head (Supplementary Figure S7). When the SINE-homologous region was used to probe the Genbank nr/nt database, positive hits were scored against fish genomes: all retrieved fragments covered entirely the SINE-homologous region and are, in fact, part of SINE elements. These SINEs were retrieved from 19 bony fish genomes, including *T. rubipes*. For 16 out of 18 newly recovered SINEs it was possible to reconstruct a full-length consensus sequence, while for two of them the RNA-related head was lacking (Supplementary Figure S9; Supplementary Data S2). The similarity between *T. rubipes* Ac1 and SINEs isolated from other fishes spanned the entire length of the sequence (head+body+tail); the Jukes–Cantor divergence ranged from 14.2% (Ac1 vs. *Larimichthys crocea*) to 31.5% (Ac1 vs. *Erpetoichthys calabaricus*).

**Figure 4:**
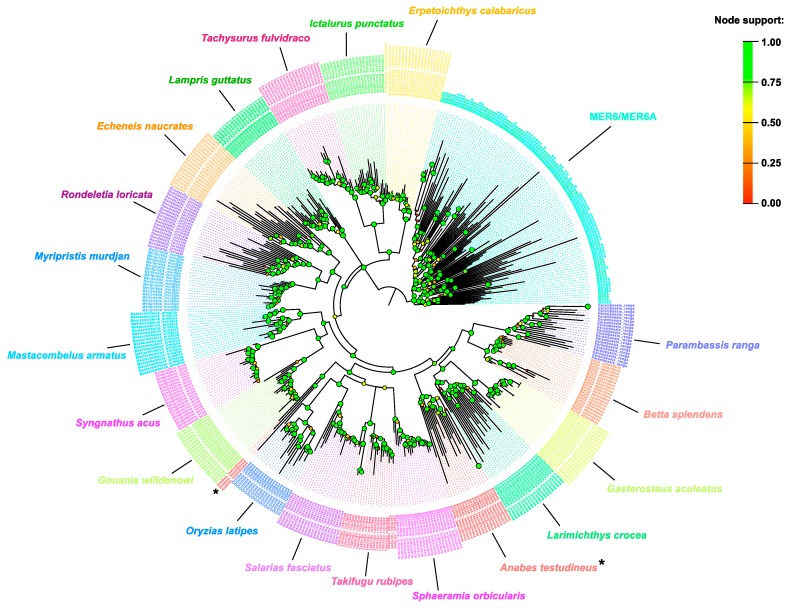
Maximum likelihood tree calculated on 477 bony fish V-SINE sequences and MER6 elements, using the Jukes–Cantor+CAT model. Coloured dots at nodes indicate the support value, as per upper-right legend. The name of fish species from which V-SINEs have been isolated are also reported near the relative cluster. Asterisks mark non-monophyletic *Anabas testudineus* Ac1-like SINEs.

To compare these new SINEs with the homologous region within MER6 and MER6A elements, up to 20 sequence per fish species were aligned together with 50 MER6 and 50 MER6A elements, sampled randomly from the main dataset, and used to build a maximum likelihood phylogenetic tree (Figure 4; Supplementary Figure S10). From the analysis of the 477 sequences, two main supported clusters emerged: one including fishes’ SINE sequences and one including MER6 homologous regions. Moreover, while within the latter group no clustering structure was evident, the SINE sequences are partitioned into species-specific clades, with the only exception of those from the climbing perch *Anabas testudineus*. Overall, the average Jukes–Cantor divergence between the homologous regions of isolated fish SINEs and of MER6 was 39.2%.

A further phylogenetic analysis has been carried out considering only the V HCD from the 477 bony fish V-SINE sequences and MER6 elements (Supplementary Figure S11). Similar to the previous analysis, MER6 HCDs were clustered separately with respect to V-SINEs HCDs, the latter being clustered according to the species origin.

The selected 85 representative genomes were further searched with SINE consensus sequences: besides significant BLAST alignments with MER6 copies, other similarities were not found in mammalian, birds or reptiles. On the other hand, as expected, BLAST significant alignments were obtained with the V HCD from V-SINEs found in anuran genomes.

## 3. Discussion

In this work, the first detailed genome-wide analysis of MER6 and MER6a non-autonomous DNA-transposons is reported. Although known so far as the unique mobile element carrying the V conserved domain in mammals (and in amniote in general) [10,16], the evolution and genomic dynamics of these elements have never been investigated.

**Figure 5:**
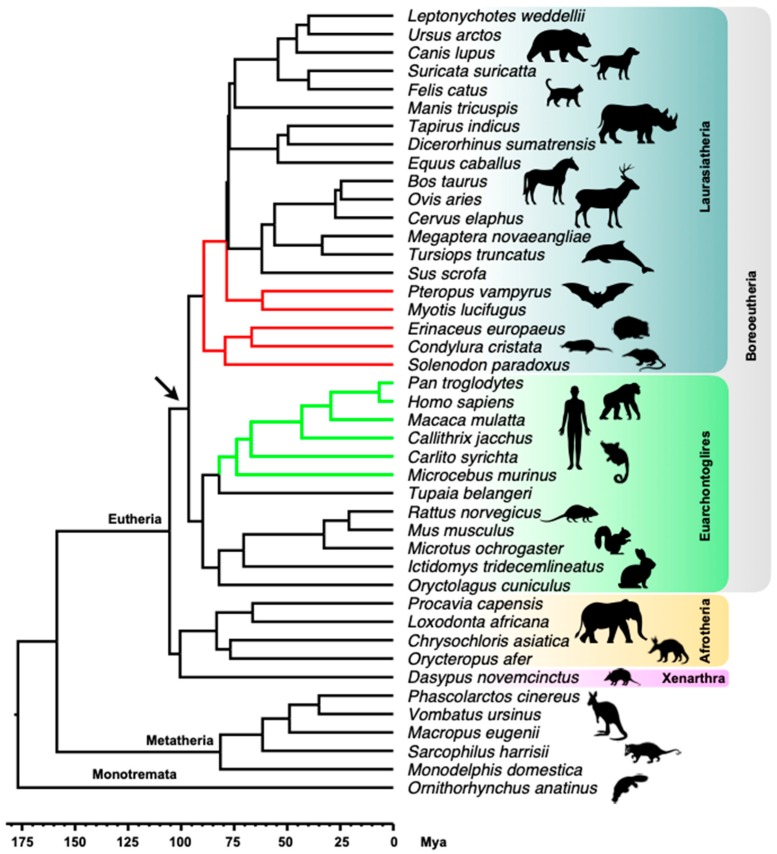
Schematic drawing of mammalian time tree, showing the MER6 elements distribution. Green branches correspond to MER6 and MER6A distribution, coincident with primates. Red branches indicate lineages were only MER6A has been retrieved (bats and eulipotyphlans lineages). The arrow marks where MER6 elements putatively originated. High-level taxonomy is reported on the right of the tree.

MER6 and MER6A have been isolated for the first time in *H. sapiens* and later labelled as primate-specific elements [26,28]. Data presented here depict a far wider distribution for the variant MER6A, which is also present in bats (order Chiroptera) and in eulipotyphlans (order Eulipotyphla). Looking at mammalian phylogeny (Figure 5), MER6A was present in two different lineages: the Euarchontoglires, to which primates belong, and the Laurasiatheria, which includes bats and eulipotyphlans. The differential distribution of MER6 and MER6A suggests that their divergence could date back to 96 Million years ago (Mya) (C.I. = 91–102 Mya), when the two mammalian clades split, and that in the Laurasiatheria lineage only MER6A survived. Moreover, within Laurasiatheria, the element further diversified, as suggested by the slightly different sequence structure in eulipotyphlan genomes. On the other hand, MER6A was not found in non-primates euarchontoglires and/or in other laurasiatherian lineages (Figure 5). In their evaluation of the timing of DNA transposon activity in the human genome, Pace and Feschotte [28] calculated the age of MER6 and MER6A as comprised between 50 Mya and 70 Mya, roughly corresponding to the initial amplification wave of Tc1/Mariner elements. Although TEs’ age estimates based on sequence divergence should be taken cautiously, because of possible bias and variation in substitution rates, it is interesting to note that considering the split at 96 Mya the substitution rate experienced by MER6 elements varies between around 2.1 × 10^−9^ (MER6) and 2.4 × 10^−9^ (MER6A) substitutions/site/year. Notably, these estimates overlap the neutral nucleotide substitution rates estimated in mammals, 2.2 × 10^−9^ substitutions/site/year [29], or in bats, 2.37 × 10^−9^ substitution/site/year [30]. Therefore, considering the phylogenetic distribution of these elements and the concordance with rough estimates of substitution rates, it is likely that the main activity of MER6 elements dates back to the origin of the Boreoeutheria crown group (Figure 5). As an alternative hypothesis, which might reconcile the taxonomic distribution and the suggested later activity [28], it could be suggested that MER6A might have been acquired in the early Laurasiatheria lineage by horizontal transfer. TEs’ horizontal transfers in mammals are quite rare, although some of these events, involving hAT, piggyBac and Tc1/Mariner elements, have been scored in vespertilionid bats (which include the presently analysed *M. lucifugus*) [31]. On the other hand, the separation between Eulipotyphla and Chiroptera, which both harbour MER6A, occurred between 81 Mya and 96 Mya, earlier than previously estimated repeats’ age of 50–70 Mya [28]. This would suggest, therefore, that multiple horizontal transfers might have occurred which appears, though, even more unlikely. It is to be noted, finally, that some technical differences may also explain the difference observed between Pace and Feschotte’s age estimates [28] and those provided in the present analysis. Their age estimates have been calculated with a different evolutionary model, using substitution rates estimated on the taxonomic distribution of transposons’ orthologous insertions and nested insertions within primate-specific retrotransposons [28]. However, their comparative genomics analysis was more limited than that presently reported and did not include Eulipotyphla and Chiroptera [28]: thus, they may have obtained biased substitution rate estimates. Moreover, Pace and Feschotte did not rule out the possibility that some of the analysed transposon families were actually older and that they were analysing just primate-specific transposon clades [28].

The phylogenetic analysis of the 8588 repeat sequences failed to retrieve a meaningful clustering pattern, i.e., based on host species (or on other taxonomic level) or on repeats variant (MER6 vs. MER6A). The use of either the Jukes–Cantor or the GTR models, the latter being more parametrized than the former, does not result in different clustering pattern, suggesting that the model choice does not significantly affect the analysis. The lack of phylogenetic structure is even more striking considering the structural difference between MER6A elements from bats and eulipotyphlans. In this regard, the absence of the phylogenetic signal could be explained in different, alternative ways. Long-term inactivity could lead to random mutations accumulation on existing TEs copies blurring the phylogenetic signal. In the present analysis there was no evidence of recent activity: in fact, if the calculated substitution rate is applied on the younger copies found among all genomes (>12% of Jukes–Cantor divergence from the consensus), it could be obtained a rough age estimate for the end of MER6 elements wave at about 54.5 Mya. Generally speaking, DNA elements in mammalian genomes are very little represented and, in agreement with the present rough estimate, previous data indicated that their activity ceased almost completely in the anthropoid lineage around 40 Mya [28]. It is to be noted, though, that clear signs of recent activity have been found in the primate *Microcebus murinus* and the microbat *Myotis lucifugus* genomes, even if this evidence refers to different DNA elements than MER6 [30,32,33,34]. An alternative view is that some form of selection is acting on these repeats, maintaining a substantial homogeneity, but the extent of variability scored among their sequences actually suggests the opposite. Overall, data presented here suggest that MER6 and MER6A were active during the early phase of Boreoeutheria diversification, and that the two repeats differentially invaded mammalian genomes.

**Figure 6:**
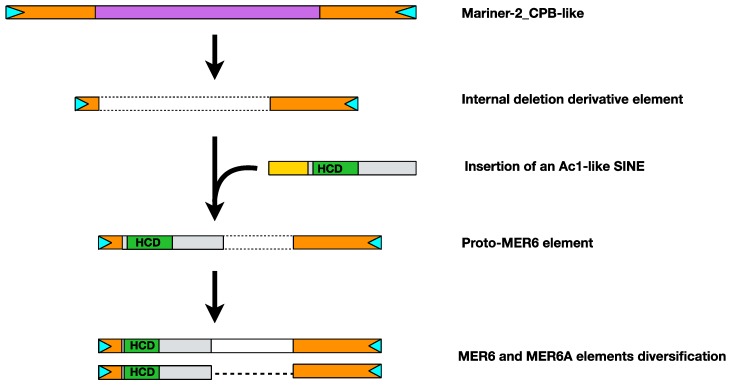
Stepwise origin of MER6 elements. The original Tc1/Mariner element, homologous to Mariner-2_CPB, lost the internal domain containing the transposase coding sequence (purple box) producing an internal deletion derivative MITE element. Then, an Ac1-like short interspersed element (SINE) inserted in the MITE (HCD: V highly conserved domain). The new composite MITE, then, diversified into MER6 and MER6A elements.

The anatomical dissection of MER6 elements structure further depicts a complex scenario for their origin and evolution. Based on terminal inverted repeats similarity, the parental autonomous Tc1/Mariner element was identified as the Mariner-2_CPB element from the genome of the turtle *C. picta*. Moreover, also the source of the V HCD was identified, as within MER6 elements a sequence fragment homologous to a nearly full-length fish-specific V-SINE, namely Ac1, was found. These evidences suggest that MER6 emerged first as internal deletion derivative of a Tc1/Mariner element homologous to Mariner-2_CPB and then an Ac1-like element retro-transposed within the new MITE (Figure 6). 

While the chimeric origin of a TE is not unexpected [35,36], the absence of MER6 elements parental components from amniote genomes is striking. In fact, the possible chimeric origin clearly requires that both the Mariner and SINE component were simultaneously present in the same genome at the moment of the chimera assembly. On the other hand, this element, or any other related element, was not found in the mammalian genome (with the exception of obvious sequence similarities at the transposase protein coding region level). The taxonomic distribution of MER6 elements suggests that they most likely originated within the ancestral Boreoeutheria lineage or, at least, during the differentiation of Eutheria (Figure 5). However, it cannot be excluded that they just went extinct in the other mammalian lineages, as well as in bird and other reptiles. In this instance, a different evolutionary scenario could be that both the Tc1/Mariner and the SINE elements were present in the reptile genome, where the ancestral MER6 sequence was assembled. However, Ac1-like elements, or even the presence of the V HCD hosted in other SINE families, were not found either in the turtle or in other reptile genomes. Yet, the clear-cut divergence between Ac1-like SINEs and their homologous region within MER6 elements points out either to an ancestral split, before the SINEs replication wave occurred in bony fishes, or to a past horizontal transfer event from a yet unidentified fish species to the early amniote lineage.

Although the present analysis cannot provide direct evidence of the origin of MER6 elements, having determined the structural original components of these MITEs highlights a unique, complex evolutionary history that developed across vertebrates.

It is worth noting that about the 40% of MER6 elements insertions have been scored within genes or in their flanking regions (±5000 bp) in all assayed genomes, with the exception of *C. syrichta* which has a significantly lower proportion of insertions within genic regions. This difference is difficult to explain without any *ad hoc* explanation, like some selective pressure preventing the conservation of MER6 element insertions in this particular genome. On the other hand, it cannot be excluded a technical bias. The *C. syrichta* genome is, in fact, by far the most fragmented among those presently analysed (Supplementary Table S2): therefore, it is likely that several selected flanking regions (and, possibly, also some genes) could have been picked up only partially, at variance with more contiguous genomes. In addition to the *C. syrichta* genome, though, the similar proportion of MER6 elements insertions across primates, chiropterans and eulipotyphlans appears to point out the absence of differential selective pressure against or favouring the insertion within genes. A more detailed analysis on the presence of MER6 elements insertions within transcripts revealed several mRNAs and lncRNAs including these MITEs. Interestingly, MER6-homologous regions constitute a portion of the protein coding region in two mRNAs. This suggests that MER6 sequences underwent exaptation, an event known to produce evolutionary novelties and already observed for both DNA transposons and retrotransposons (reviewed in [2]). However, in addition to these two examples, the remaining MER6 elements insertions were found in the 5’ and 3’ UTRs. In a V-SINEs survey in fish genomes, it has been found that these elements can be frequently found in mRNAs and, therefore, it has been hypothesized that the V HCD could have some regulatory function [23]. A similar hypothesis has been also suggested for CORE and Deu HCDs found in the elephant shark’s SINEs [24]. It is, therefore, possible that the presence of MER6/MER6A insertions in mRNAs could have a similar role.

The V HCD has never been found in amniote genomes as a part of active V-SINE elements [10,16]: in fact, the V-SINE included in MER6 MITEs lost its head module, which prevent the transcription and, therefore, the independent replication of the retroelement itself [4]. The replication of the analysed MITEs helped to distribute the V HCD within primate, chiropteran and eulipotyphlan lineages, contributing to its spreading within these genomes. On the other hand, in the majority of analysed mammalian lineages, including primates, DNA elements are generally less represented and generally less active [28]. Therefore, the extent of genomic distribution of V HCD within mammalian genomes appears more limited that those observed in non-amniotic genomes.

A metazoan-wide survey of HCDs distribution, including the V one, suggested that their high conservation cannot be attributed to horizontal transfer events: on the contrary, phylogenetic and age vs. divergence analyses indicated a pattern of vertical inheritance [16]. Our data on the taxonomic distribution and evolutionary history of MER6 MITEs seem to confirm this pattern and provide evidence of the widespread occurrence and conservation of the V HCD in three different mammalian lineages. The presence of MER6 insertions within genes and within mRNAs could suggest some functional roles that are worth to be better investigated in future analyses. Overall, data presented here clearly show how complex interplays between different TEs could give raise to new forms of elements with the potential to impact on the host genome and cell functions.

## 4. Materials and Methods

Consensus sequences of MER6 and MER6A were downloaded from Repbase Update [25] (accessed on January 2019). They were used as query sequences to probe 85 representative tetrapod genomes (43 mammalians, 21 birds, 13 reptiles and eight amphibians; Supplementary Table S3) with BLAST [37], using the *blastn* algorithm with default parameters and *e*-value <1 × 10^−10^. Standard BLAST output files were transformed into FASTA alignment files through Mview v. 1.6 [38] and the query coverage was calculated as the number of nucleotides aligned in each position using the *profile* function of the R library seqinr [39].

MER6 and MER6A copies were obtained after running RepeatMasker [40] on genome assemblies, with default parameters. Only positive hits covering >50% of the query sequence were taken into account, in order to avoid overlapping similarities between MER6 and MER6A. Then, to refine the copy number estimation, the Onecodetofindthemall.pl script [41] was used to merge adjacent fragments and isolate each copy from the respective genome (using the built-in *--fasta* function).

The putative parental autonomous element was searched using BLAST, with parameter as described above, in the Tc1/Mariner collection found in RepBase (accessed on January 2019). Moreover, the internal region carrying the V HCD was compared with the SINE collection found in RepBase (accessed on January 2019) using the same search method.

SINEs matching the MER6 SINE-homologous region were retrieved by searching into Genbank nucleotide database (*nt/nr*; accessed on August 2019) using a BLAST search as described above.

Sequence alignments were performed using MAFFT v.7 [42], using the FFT-NS-1 parameter set. The analysis of sequence divergence from the relative species-specific consensus sequence was used to tentatively estimate the MITEs activity through time, considering the divergence a proxy for time since the insertion. According to this principle, highly divergent copies from the consensus indicate past activity, while less divergent copies would indicate a more recent activity [43]. The divergence was estimated using the Jukes–Cantor nucleotide substitution model, which accounts for possible multiple substitutions. Phylogenetic analyses were carried out using FastTree v. 2.1.11 [44] with default parameters, setting either the Jukes–Cantor or the GTR (General Time Reversible) nucleotide substitution models with the CAT approximation for the among site variation.

MER6 elements within genes ±5000 bp flanking regions were found by analysing the overlap between insertions and gene annotations, obtained from Genbank, through BEDTools v. 2.17 [45] using the *intersectBed* function.

Mammalian phylogeny and time estimates of cladogenetic events were obtained from TimeTree.org (last accessed on August 2019) [46]. The MER6 elements substitution rate was derived from the formula *T* = *D*/*s*, where *T* is the time of the cladogenetic event, *D* is the divergence from the consensus and *s* is the substitution rate [47].

## Figures and Tables

**Table 1:** Summary statistics of MER6 and MER6A assayed genomes.

Species	MER6			MER6A		
	No. of Insertions	Genome Coverage (%)	Divergence from the Consensus (%)	No. of Insertions	Genome Coverage (%)	Divergence from the Consensus (%)
*Homo sapiens*	428	0.01	19.2	545	0.01	19.0
*Pan troglodytes*	411	0.01	19.2	538	0.01	19.1
*Callithrix jacchus*	364	0.009	21.9	482	0.009	21.6
*Macaca mulatta*	422	0.01	20.2	544	0.01	19.8
*Carlito syrichta*	428	0.009	22.9	648	0.01	22.1
*Microcebus murinus*	385	0.011	19.8	544	0.012	19.3
*Pteropus vampyrus*				873	0.023	18.6
*Myotis lucifugus*				544	0.015	19.2
*Condylura cristata*				425	0.012	30.2
*Erinaceus europaeus*				136	0.002	34.9
*Solenodon paradoxus*				871	0.022	27.6

**Table 2:** Occurrence of BLAST significant alignments of MER6 and MER6A in transcriptomes.

Species	mRNA				lncRNA	Miscellaneous RNA
	Total	5′ UTR	CDS	3′ UTR		
*Homo sapiens*	39	15	--	24	40	4
*Pan troglodytes*	33	6	1	26	11	4
*Callithrix jacchus*	9	2	1	6	5	2
*Macaca mulatta*	12	3	--	9	7	--
*Carlito syrichta*	1	--	--	1	1	--
*Microcebus murinus*	23	18	--	5	17	2
*Pteropus vampyrus*	34	10	--	24	11	9
*Myotis lucifugus*	16	4	--	12	--	--
*Condylura cristata*	3	--	--	3	--	--
*Erinaceus europaeus*	--	--	--	--	--	--

## Supplementary Materials

Supplementary materials can be found at https://www.mdpi.com/1422-0067/20/22/5607/s1.

## Abbreviations

TE	Transposable elements
SINE	Short interspersed elements
MITE	Miniature inverted-repeat transposable element
HCD	Highly conserved domain
mRNA	Messenger RNA
miRNA	Micro-RNA
lncRNA	Long non-coding RNA
tRNA	Transfer RNA
UTR	Untranslated region
GTR	General Time Reversible

## References

[1] Wicker T., Sabot F., Hua-Van A., Bennetzen J.L., Capy P., Chalhoub B., Flavell A., Leroy P., Morgante M., Panaud O. (2007). A unified classification system for eukaryotic transposable elements. Nat. Rev. Genet..

[2] Bourque G., Burns K.H., Gehring M., Goburnova V., Seluanov A., Hammel M., Imbeault M., Izsvák Z., Levin H.L., Macfarlan T.S. (2018). Ten things you should know about transposable elements. Genome Biol..

[3] McClintock B. (1929). A Cytological and Genetical Study of Triploid Maize. Genetics.

[4] Kramerov D.A., Vassetzky N.S. (2011). Origin and evolution of SINEs in eukaryotic genomes. Heredity.

[5] Feschotte C., Pritham E.J. (2007). DNA Transposons and the evolution of eukaryotic genomes. Annu. Rev. Genet..

[6] Chalopin D., Naville M., Plard F., Galiana D., Volff J.-N. (2015). Comparative analysis of transposable elements highlights mobilome diversity and evolution in vertebrates. Genome Biol. Evol..

[7] Kojima K.K. (2018). LINEs Contribute to the origins of middle bodies of SINEs besides 3′ tails. Genome Biol. Evol..

[8] Luchetti A., Mantovani B. (2013). Conserved domains and SINE diversity during animal evolution. Genomics.

[9] Gilbert N., Labuda D. (1999). CORE-SINEs: Eukaryotic short interspersed retroposing elements with common sequence motifs. Proc. Natl. Acad. Sci. USA.

[10] Ogiwara I., Miya M., Ohshima K., Okada N. (2002). V-SINEs: A new superfamily of vertebrate SINEs that are widespread in vertebrate genomes and retain a strongly conserved segment within each repetitive unit. Genome Res..

[11] Nishihara H., Smit A.F., Okada N. (2006). Functional noncoding sequences derived from SINEs in the mammalian genome. Genome Res..

[12] Nishihara H., Plazzi F., Passamonti M., Okada N. (2016). MetaSINEs: Broad distribution of a novel SINE superfamily in animals. Genome Biol. Evol..

[13] Akasaki T., Nikaido M., Nishihara H., Tsuchiya K., Segawa S., Okada N. (2010). Characterization of a novel SINE superfamily from invertebrates: “Ceph-SINEs” from the genomes of squids and cuttlefish. Gene.

[14] Piskurek O., Jackson D.J. (2011). Tracking the ancestry of a deeply conserved eumetazoan SINE domain. Mol. Biol. Evol..

[15] Matetovici I., Sajgo S., Ianc B., Ochis C., Bulzu P., Popescu O., Damert A. (2016). Mobile element evolution playing jigsaw-SINEs in gastropod and bivalve mollusks. Genome Biol. Evol..

[16] Luchetti A., Mantovani B. (2016). Rare horizontal transmission does not hide long-term inheritance of SINE highly conserved domains in the metazoan evolution. Curr. Zool..

[17] Gilbert N., Labuda D. (2000). Evolutionary inventions and continuity of CORE-SINEs in mammals. J. Mol. Biol..

[18] Deragon J.-M., Grandbastien M.-A., Casacuberta J.M. (2012). SINE exaptation as cellular regulators occurred numerous times during eukaryote evolution. Plant Transposable Elements.

[19] Bejerano G., Lowe C.B., Ahituv N., King B., Siepel A., Salama S.R., Rubin E.M., Kent W.J., Haussler D. (2006). A distal enhancer and an ultraconserved exon are derived from a novel retroposon. Nature.

[20] Santangelo A.M., de Souza F.S., Franchini L.F., Bumaschny V.F., Low M.J., Rubinstein M. (2007). Ancient exaptation of a CORE-SINE retroposon into a highly conserved mammalian neuronal enhancer of the proopiomelanocortin gene. PLoS Genet..

[21] Sasaki T., Nishihara H., Hirakawa M., Fujimura K., Tanaka M., Kokubo N., Kimura-Yoshida C., Matsuo I., Sumiyama K., Saitou N. (2008). Possible involvement of SINEs in mammalian brain formation. Proc. Natl. Acad. Sci. USA.

[22] Nakanishi A., Kobayashi N., Suzuki-Hirano A., Nishihara H., Sasaki T., Hirakawa M., Sumiyama K., Shimogori T., Okada N. (2012). A SINE-derived element constitutes a unique modular enhancer for mammalian diencephalic Fgf8. PLoS ONE.

[23] Scarpato M., Angelini C., Cocca E., Pallotta M.M., Morescalchi M.A., Capriglione T. (2015). Short interspersed DNA elements and miRNAs: A novel hidden gene regulation layer in zebrafish?. Chromosome Res..

[24] Luchetti A., Plazzi F., Mantovani B. (2017). Evolution of two short interspersed elements in *Callorhinchus milii* (Chondrichthyes, Holocephali) and related elements in sharks and the coelacanth. Genome Biol. Evol..

[25] Bao W., Kojima K.K., Kohany O. (2015). Repbase update, a database of repetitive elements in eukaryotic genomes. Mob. DNA.

[26] Smit A.F.A., Riggs A.D. (1996). *Tiggers* and DNA transposon fossils in the human genome. Proc. Natl. Acad. Sci. USA.

[27] Feschotte C., Zhang X., Wessler S.R., Craig N., Craigie R., Gellert M., Lambowitz A. (2002). Miniature inverted-repeat transposable elements (MITEs) and their relationship with established DNA transposons. Mobile DNA II.

[28] Pace J.K., Feschotte C. (2007). The evolutionary history of human DNA transposons: Evidence for intense activity in the primate lineage. Genome Res..

[29] Kumar S., Subramanian S. (2002). Mutation rates in mammalian genomes. Proc. Natl. Acad. Sci. USA.

[30] Ray D.A., Feschotte C., Pagan H.J.T., Smith J.D., Pritham E.J., Arensburger P., Atkinson P.W., Craig N.L. (2008). Multiple waves of recent DNA transposon activity in the bat, *Myotis lucifugus*. Genome Res..

[31] Platt R.N., Vandewege M.W., Ray D.A. (2018). Mammalian transposable elements and their impacts on genome evolution. Chromosome Res..

[32] Pritham E.J., Feschotte C. (2007). Massive amplification of rolling-circle transposons in the lineage of the bat *Myotis lucifugus*. Proc. Natl. Acad. Sci. USA.

[33] Ray D.A., Pagan H.J.T., Thompson M.L., Stevens R.D. (2007). Bats with hats: Evidence for recent DNA transposon activity in genus *Myotis*. Mol. Biol. Evol..

[34] Pagan H.J., Smith J.D., Hubley R.M., Ray D.A. (2010). PiggyBac-ing on a primate genome: Novel elements, recent activity and horizontal transfer. Genome Biol. Evol..

[35] Malik H.S., Eickbush T.H. (2001). Phylogenetic analysis of ribonuclease H domains suggests a late, chimeric origin of LTR retrotransposable elements and retroviruses. Genome Res..

[36] Ostertag E.M., Goodier J.L., Zhang Y., Kazazian H.H. (2003). SVA elements are nonautonomous retrotransposons that cause disease in humans. Am. J. Hum. Genet..

[37] Altschul S.F., Gish W., Miller W., Myers E.W., Lipman D.J. (1990). Basic local alignment search tool. J. Mol. Biol..

[38] Brown N.P., Leroy C., Sander C. (1998). MView: A Web compatible database search or multiple alignment viewer. Bioinformatics.

[39] Charif D., Lobry J., Bastolla U., Porto M., Roman H., Vendruscolo M. (2007). SeqinR 1.0-2: A contributed package to the R project for statistical computing devoted to biological sequences retrieval and analysis. Structural Approaches to Sequence Evolution: Molecules, Networks, Populations.

[40] Smit A.F.A., Hubley R., Green P. (2013–2015). RepeatMasker Open-4.0. http://www.repeatmasker.org.

[41] Bailly-Bechet M., Haudry A., Lerat E. (2014). “One code to find them all”: A Perl tool to conveniently parse RepeatMasker output files. Mob. DNA.

[42] Katoh K., Standley D.M. (2013). MAFFT Multiple Sequence Alignment Software Version 7: Improvements in performance and usability. Mol. Biol. Evol..

[43] Huang C.R.L., Burns K.H., Boeke J.D. (2012). Active transposition in genomes. Annu. Rev. Genet..

[44] Price M.N., Dehal P.S., Arkin A.P. (2010). FastTree 2—Approximately maximum-likelihood trees for large alignments. PLoS ONE.

[45] Quinlan A.R., Hall I.M. (2010). BEDTools: A flexible suite of utilities for comparing genomic features. Bioinformatics.

[46] Hedegs S.B., Marin J., Suleski M., Paymer M., Kumar S. (2015). Tree of life reveals clock-like speciation and diversification. Mol. Biol. Evol..

[47] Kapitonov V.V., Jurka J. (2003). Molecular paleontology of transposable elements in the *Drosophila melanogaster* genome. Proc. Natl. Acad. Sci. USA.

