# Supplementary material for: Riding the Wave: The SINE-Specific V Highly-Conserved Domain Spread into Mammalian Genomes Exploiting the Replication Burst of the MER6 DNA Transposon
Source: Int J Mol Sci. 2019 Nov 9;20(22):5607. doi: 10.3390/ijms20225607 (PMC6887750; doi:10.3390/ijms20225607)
Supplement: Supplementary file 1 [file ijms-20-05607-s001.pdf]

```
                              1
V_HCD                         ---------- ---------- ---------- ---------- ---------- ----------
Racla_(Rana_clamitans)        -----CGGCA CAGTGGCGTA GTGGTTAGCA CTYTCGCC-T AGCAGTAAGA AGGGTCACTG
Nanorana_parkeri_VSINE        GTGGGCAGCA CAGTGGCGTA -TGGTTAGCA CTTCTGCC-T TGCAGCACTG GGGGTCCCTG
Pyxicephalus_adspersus        CAGGGCAGCA CGGTGGCTCA GTGGTTAGCA CTCTGGCCTT TGCAGCGCTA --GGTCCCAG
Rana_catesbeiana_VSINE        GTGGGCGGCA CAGTGGTGTA -TGGATAGCA CTTTCGCC-T AGCAGTAAGA AGGGTCGCTG
Rhinella_marina_VSINE         CAGGGCAGCA CGGTGGCTCA GTGGTTAGCA CTGGTGCC-T TGCAGCGCTG -GGGTCCTGG

                             61
V_HCD                         ---------- ---------- -CTTTCTGTG TGGAGTTTGC ATGTTCTCCC TGTGTCTGCG
Racla_(Rana_clamitans)        GTTCGAATCC CGACCACGAC ACTACCTGCC TGGAGTTTGC ATGTTCTCCC TGTGCCTGCG
Nanorana_parkeri_VSINE        GTTCGAATCC CGGCCAGGAC ACTATCTGCA AGGAGTTTGC ATGTTCTCCC TGTGCTTGCG
Pyxicephalus_adspersus        GTTCGAATCT CAGCCAGGAC ACTATCTGCA TGGAGTTTGC AGGTTCTCCC CGTGTTTGCG
Rana_catesbeiana_VSINE        GTTCGAATCC CAACCACGAC ACTACCTGCC TGGAGTTTGC ATGTTCTCCC TGTGCCTGCG
Rhinella_marina_VSINE         GTTTGAATCC GACCAAGGAC AACATCTGCA TGGAGTTTGT ATGTTCTCCC TGTGTTTGCG

                            121
V_HCD                         TGGGTTTCCT CCGGGTGCTC CGGTTTCCTC CCACAGTCCA AAGACATGCA GGWTATAGGT
Racla_(Rana_clamitans)        TGGGTTTCCT CCGGGTACTC CGGTTTCCTC CCACACTCCA AAGACATGCT GG---TAGGT
Nanorana_parkeri_VSINE        TGGGTTTCCT CCGGGTACTC CGGTTTCCTC CCACACTCCA AAGACATGCT GG---TAGGT
Pyxicephalus_adspersus        TGGGTTTCCT CCGGGTACTC CGGTTTCCTC CCACATTCCA AAAACATGCA GT---TAGGT
Rana_catesbeiana_VSINE        TGGGTTTCCT CCGGGTACTC CGGTTTCCTC CCACACTCCA AAGACATGCT GG---TAGGT
Rhinella_marina_VSINE         TGGGTTTCCT CCGGGTACTC CGGTTTCCTC CCACACTCCA AAGACATACT GA--------

                            181
V_HCD                         TAATTGGACA TTCT-----A ATTGCCCMTA ---------- ---------- ----------
Racla_(Rana_clamitans)        TAATTGGATC CTGTCT--AA ATTGGCCCTA ---------- ---------- GTATGTATGA
Nanorana_parkeri_VSINE        TAATTGGCTC CTGTCT--AA ATTGGCCCTA GTATGTGTGT GTGTGTATGT GTATGTATGC
Pyxicephalus_adspersus        TAATTGGCTT CCCCCTAAAA ATTGACCTTA GACTGTATTA ATG------- --ACATATGA
Rana_catesbeiana_VSINE        TAATTGGATC CTGTCT--AA ATTGTCCCTA ATATGTGTGT GTGTGTATGT GTATGTATGA
Rhinella_marina_VSINE         ---------- ---------- ---------- ---------- ---------- ----------

                            241
V_HCD                         ---------- ---------- ---------- ---------- ---------- ----------
Racla_(Rana_clamitans)        ATGTGAGTTA GGGACCTTAG ATTGTAAGCT CCTT--GAGG GCAGGGACTG ATGTGAATGT
Nanorana_parkeri_VSINE        ATGTGAGATA GGGACCTTAG ATTGTAAGCT CCTT--GAGG GCAGGGACTG ATGTGAATGT
Pyxicephalus_adspersus        CTATGG--TA GGGACATTAG ATTGTGAGCC CCTTTGAGGG ACAGCTAGTG ACATGACTAT
Rana_catesbeiana_VSINE        ATGTGAGTTA GGGACCTTAG ATTGTAAGCT CCTT--GAGG GTAGGGACTG ATGTGAATGT
Rhinella_marina_VSINE         --------TA GGGAACTTAG ATTGTGAGCC CCATT-GGGG ACAGT--TTG ATGCTAATGT

                            301
V_HCD                         ---------- ---------- ---------- ---------- ---------- ----------
Racla_(Rana_clamitans)        ATAATATATA TGTAAAGCGC TGCGTAAATT GACGGCGCTA TATAAGTACC T-GAAATAAA
Nanorana_parkeri_VSINE        GCAATGTATA TGTAAAGCGC TGCGTAAATT GTTGGCGCTA TATAAATACC T-GTAATAAA
Pyxicephalus_adspersus        GGACTTT--- -GTACAGCGC TGCGTAATAT GTTGGCGCTA TATAAATACT GTTTAATAAT
Rana_catesbeiana_VSINE        ACAATGTATA TGTAAAGCGC TGCGTAAATT GACGGCGCTA TATAAGTACC T-GAAATAAA
Rhinella_marina_VSINE         ---CTGTA-- ---AAAGCGC TGCGGAATAT AGTAGCGCTA TATAAGTGCA TAAAAATAAA

                            361
V_HCD                         ---------- ----
Racla_(Rana_clamitans)        TAAAWAAATA AATA
Nanorana_parkeri_VSINE        TAAA------ ----
Pyxicephalus_adspersus        AATAATAATA A---
Rana_catesbeiana_VSINE        TAAA------ ----
Rhinella_marina_VSINE         TAAA------ ----
```

Suppl. Figure S1. Alignment of newly found amphibian V-SINEs with the *Racla* V-SINE and the consensus sequence of V highly conserved domain.

```
MER6          CAGCAGGTCCTCGAATAACGTCGTTTCGTTCAACGTCGTTTCGTTATAACGTTGATGAGAAAAAAAA--TCGATTCCCGG   (78)
MER6A         CAGCAGGTCCTCGAATAACGTCATTTCGTTCAACGTCGTTTCGTTATAACGTTGATGAGAAAAAAAA--TCGATTCCCGG   (78)
C. cristata   CAGCAGGTCCTCAAATAATGTCATTTTGTTCAATGTCATTTTATTATAATGTTGATGAGAAAAAAA------ATTCCTGG   (74)
E. europaeus  CAGCAGGTCCTCGAATAA--TTATTT--TTCAA-GTCATTT--TTATAATATTGATGAAAAAAAAAA--TTGATTCCCGG   (71)
S. paradoxus  CAGCAGGTCCTCAAATAATGTCATTTCATTCAATGTCATTTCATTATAATATTGATGAGAAAAAAAAA----ATTCCTGG   (76)

MER6          CCGGGGCCACTGTCTGTGTGGAGTTTGCACGTTCTCCCCATGTCTGCGTGGGTTTCTCCGGGTACTCCGGTTTCCTCCC   (158)
MER6A         CCGGGGCCACTGTCTGTGTGGAGTTTGCACGTTCTCCCCATGTCTGCGTGGGTTTCTCCGGGTACTCCGGTTTCCTCCC   (158)
C. cristata   CCAGGGCCACTGTCTGTGTGGAGTTTGCATGTTCTCCCCATGTCTGTGTGGGTTTCTCTGGGTACTCTGGTTTCCTCCC   (154)
E. europaeus  CTAGGGCCACTGTCTGTGTGGAGTTTGCACATTCTCCCCATGTCT--GTGGGTTTCTCTGGG--CTC-GGTTTCCTCCC   (146)
S. paradoxus  CCAGGGCCACTGTCTGTGTGGAGTTTGCACGTTCTCCCCATGTCTGTGTGGGTTTCTCTGGGTACTCTGGTTTCCTCCC   (156)

MER6          ACATCCCAAAGATGTGCACGTTAGGTTAATTGGCGTGTCTAMATGGTCCCAGTCTGAGTGAGTGTGGGTGTGTGTGTGAG   (238)
MER6A         ACATCCCAAAGATGTGCACGTTAGGTKAATTGGCGTGTCTACATGGTCCCAGTCTGAGTGAGTGTGGGTGTGTGTGTGAG   (238)
C. cristata   ACATCCCAAAGATGTGCATGTTAGGTTCATTGGCGTGTCTCAATTGTCCCAGTCTGAGTGAGTGTGGGTGTGT-------   (227)
E. europaeus  ACATCCCAAAGATGTGCATGTTAGGTTCACTGGTATGTCTAAATTGTCCCAGTGTGAGTGAGTGTGTGTGTGTGG-----   (221)
S. paradoxus  ACATCCCAAAGATGTGCACGTTAGGTTCATTGGTGTGTCTCAATTGTCCCAGTCTGAGTGAGTGTGTGTGTGTGTGTG--   (234)

MER6          TGCGCCCTGCGATGGGATGGCGTCCTGTCCAGGGTTGGTTCCCGCCTTGCGCCCTGAGCTGCCGGGATAGGCTCCGGCCA   (318)
MER6A         TGCGCCCTGCGATGGGATGGCGTCCTGTCCAGGGTTGGTTCCCGCCTTGTGCCCTGAGCTGCCGGGATAGGCTCCGGCCA   (318)
C. cristata   ---GCCCTG-GATGGAAGGGTGTCCTGTCCAGGGTTGGTTCCTACCTTGTGCCCTGAGCTGCCAGGATAGGCTCTGGCCA   (303)
E. europaeus  ---TCCCTGTGATGGAAGGGCATCCTGTCCAGGGTTGGTTCCTACCTTGTGCCCTGAGCTGCTAGGATAGGCTCTAGCCA   (298)
S. paradoxus  ---GCCCTGTGATGGAAGGGCGTCCTGTCCAGGGTTGGTTCCCACCTTGTGCCCTGAGCTGCTGGGATAGGCTCTGGCCA   (311)

MER6          CCCGCGACCCTGAACTGGAATAAGCGGGTTGGAAAATGAATGAATGAATGAATACAAATTATTGTAAAATAAAAATTTAT   398
MER6A         CCCGCGACCCTGAACTGGAATAATTGGGT----AAATA------------------------------------------   (362)
C. cristata   CCCGTGACCCTGAACTGGAATAAGTGGGTTGGAAAATG----AATGAATGAATACAAATT--------------------   (359)
E. europaeus  CCTGTGACCCTGAACTGGAATAAGTGGGTTGGAAAATGAATGAATGAATGAATACAAATT--------------------   (358)
S. paradoxus  CCCGTGACCCTGAACTGGAATAAGTGGGTTGGAAAATGAATGAATGAATGAATACAAATT--------------------   (371)

MER6          AAAGTATACGATAATCATACAAATGCACGACAATAAATGATGTGGTACGAAAGTGCTCAGCGAGCCCGCCATATTTGTGA   (478)
MER6A         --------------------------------------------------------------------------------   (362)
C. cristata   --------------------------------------------------------------------------------   (359)
E. europaeus  --------------------------------------------------------------------------------   (358)
S. paradoxus  --------------------------------------------------------------------------------   (371)

MER6          TTGTTTGTTTTTGAACTGCGTGGTGGTAGGAGGTGCTCCTTACAATTTTCGCTTTGCAAACATTTATTCCTTGATTTAAC   (558)
MER6A         --------------------------------------------------------------------------------   (362)
C. cristata   --------------------------------------------------------------------------------   (359)
E. europaeus  --------------------------------------------------------------------------------   (358)
S. paradoxus  --------------------------------------------------------------------------------   (371)

MER6          CCACCACCACTACGACCGCCGTCACTCACTGATTCACCAAAAATTGGGTAAATAATTATCTTACTTGTTTTTATTAATCT   (638)
MER6A         ------------------------------------------------ATTATCTTACTTGTTTTTATTAATCT   (388)
C. cristata   --------------------------------------------------------------------------------   (359)
E. europaeus  --------------------------------------------------------------------------------   (358)
S. paradoxus  --------------------------------------------------------------------------------   (371)

MER6          TTCTTAAATGTATGTATAGCTCACATTTATTTCAATGTTTAATATTAGAAGTGTTTT-GGTCTTTATTTAGAAGTTTGGT   (717)
MER6A         TTCTTAAATGTATGTATAGCTCACATTTATTTCAATGTTTAATATTAGAAGTGTTTT-GGTCTTTATTTAGAAGTTTGGT   (467)
C. cristata   ------------------------------ATTGTTTAATATTAGAAGTGTTTTGGGTCTTTATTTAGAAGTTTGGT   (406)
E. europaeus  ------------------------------ATTGTTTAATATTAGAAGTGTTTTGGGTCTTTATTTAGAAGTTTGGT   (405)
S. paradoxus  ------------------------------ATTGTTTAATATTAGAAGTGTTTTGGGTCTTTATTTAGAAGTTTGGT   (418)

MER6          GATGTTTTTGTGACCAGAAATATGCCGTAGGAACTTAACTCTTGTTTATATCAATTAGCCTATGGTAAAATTGGTTTCGT   (797)
MER6A         GATGTTTTTGTGACCAGAAATATGCCGTAGGAACTTAACTCTTGTTTATATCAATTAGCCTATGGTAAAATTGGTTTCGT   (547)
C. cristata   GATGTTTTTGTGACCAGAAATATGCCATAGGAACTTAACTCTTGTTTATATCAATTAGCCTATGGTAAAATTGGTTTCAT   (486)
E. europaeus  GATGTTTTTGTGACCAGAAATATGCTATAGGAACTTAACTCTTGTTTATATCAATTAGCCTATGGTAAAATTGGTTTTAT   (485)
S. paradoxus  GATGTTTTTGTGACCAGAAATATGCCATAGGAACTTAACTCTTGTTTATATCAATTAGCCTATGGTAAAATTGGTTTCAT   (498)

MER6          TATACGTCGTTTCGCTTAAAGTCGCAGTTTCCAAGAACCTATCGACGACGTTAAGTGAGGACTTACTG   (865)
MER6A         TATACGTCGTTTCGCTTAAAGTCGCAGTTTCCAAGAACCTATCGACGACGTTAAGTGAGGACTTACTG   (615)
C. cristata   TATATGTCATTTTGCTTAAAGTCACAGTTTCCAAGAACCTATCGATGATGTTAAGTGAGGACTTACTG   (554)
E. europaeus  TATA--TTATTTTACTTAAAGTTACAGTTTCCAAGAACCTATTAATGATATTAAGTGAGGACTTACTG   (551)
S. paradoxus  TATATATCATTTTACTTAAAGTCACAGTTTCCAAGAACCTATCGATGATGTTAAGTGAGGACTTACTG   (566)
```

Suppl. Figure S2. Alignment of consensus sequences of primate MER6 elements and MER6A variants found in eulipotyphlans (*Condylura cristata*, *Erinaceus europaeus* and *Solenodon paradoxus*).

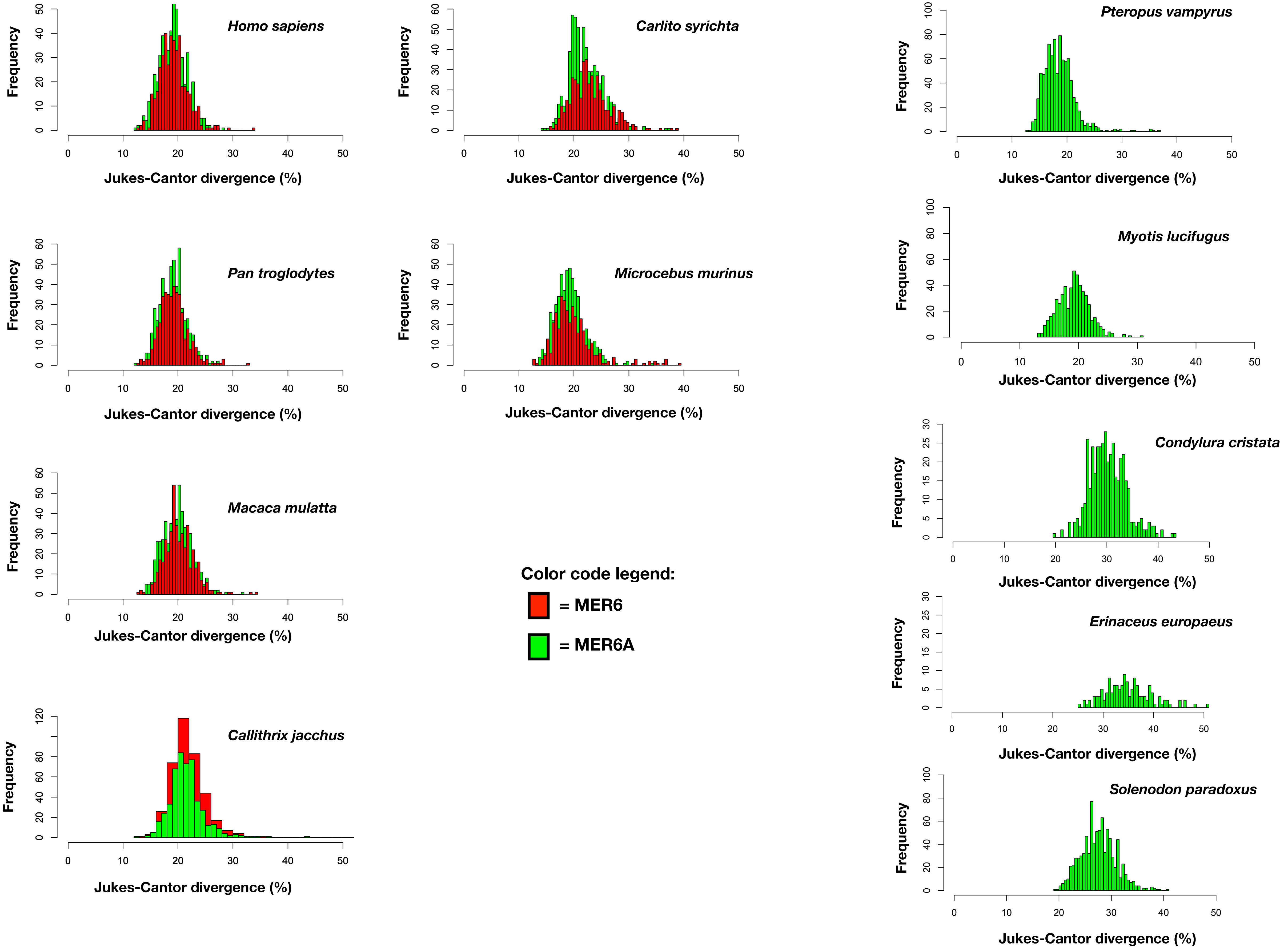

Suppl. Figure S3. MER6 elements divergence from the consensus analyses on assayed genomes.

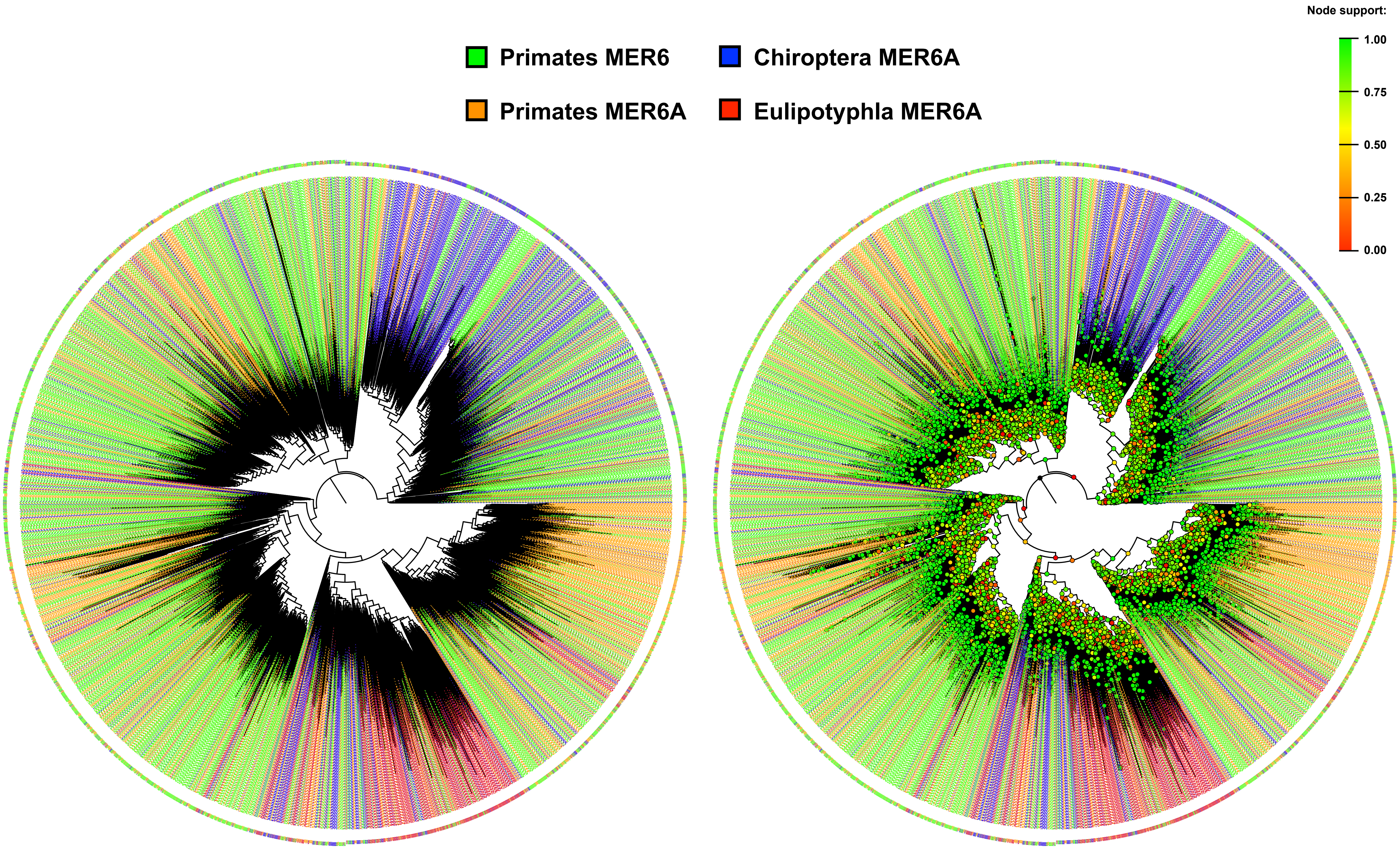

Suppl. Figure S4. Maximum likelihood phylogenetic analysis on 2,438 MER6 and the 6,150 MER6A copies isolated from primates, chiropterans and eulipotyphlans. In the left panel, the maximum Likelihood tree calculated using the GTR+CAT model without node supports for improving the graphical clarity. In the right panel, the same phylogenetic tree with node support represented by coloured dots as per upper-right legend.

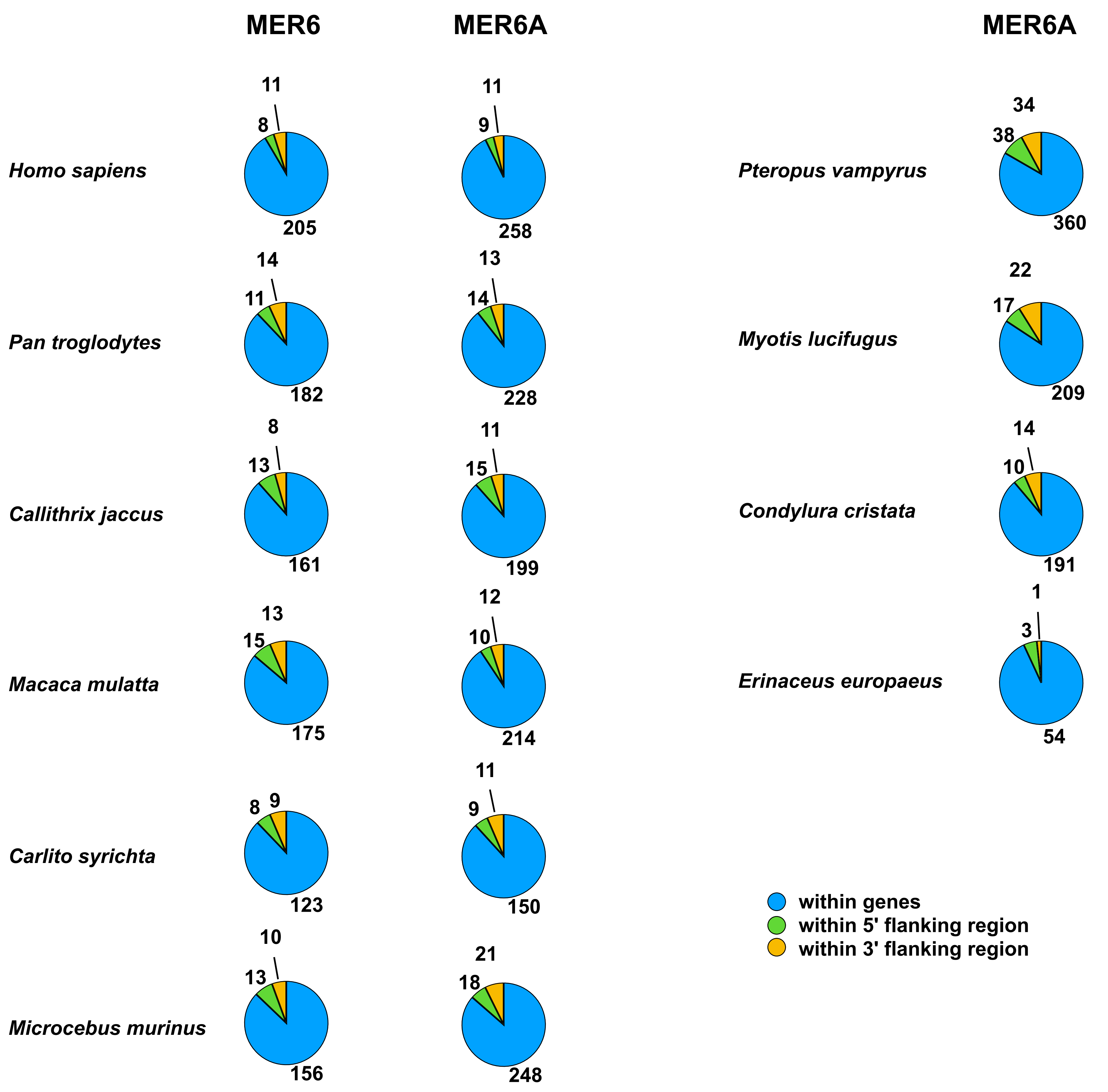

Suppl. Figure S5. Number of MER6 and MER6A insertions scored within annotated genes±5,000 bp flanking regions.

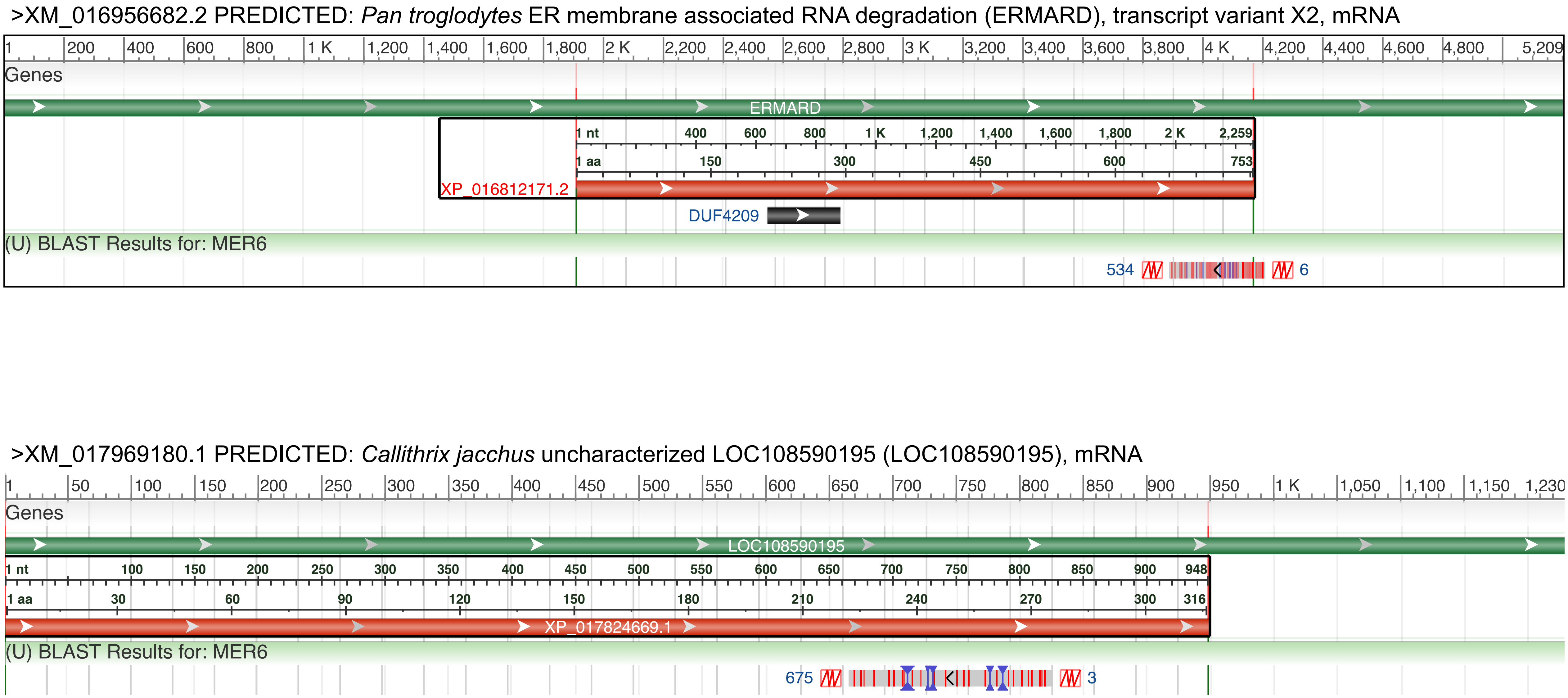

Suppl. Figure S6. Graphic view of the two BLAST results where MER6 consensus sequence significantly align with the protein coding region of two mRNAs from *Pan troglodytes* (upper panel) and *Callithrix jacchus* (lower panel), respectively. Numbers surrounding the MER6 fragment indicate the start and the end of the aligned element's region.

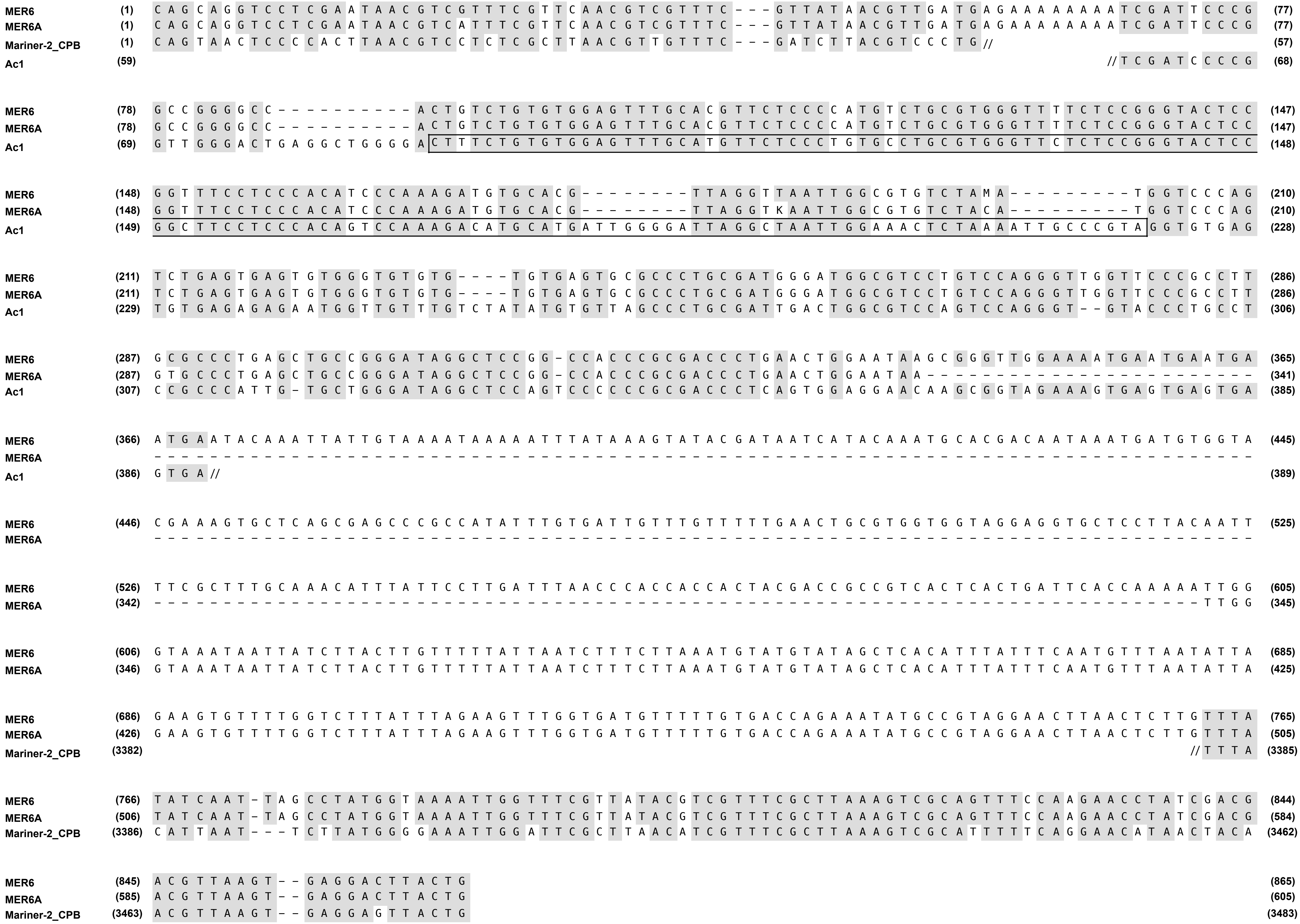

Suppl. Figure S7. Alignment of MER6 elements with homologous regions frmo turtle's Mariner-2_CPB and Fugu's Ac1 consensus sequences. Grey-shaded nucleotides indicate regions of BLAST similarity; the Ac1 V highly conserved domain is boxed. The double bar (//) indicates were the homologus region start/stop. Numbers between parentheses indicate the realtive nucleotide position of each sequence.

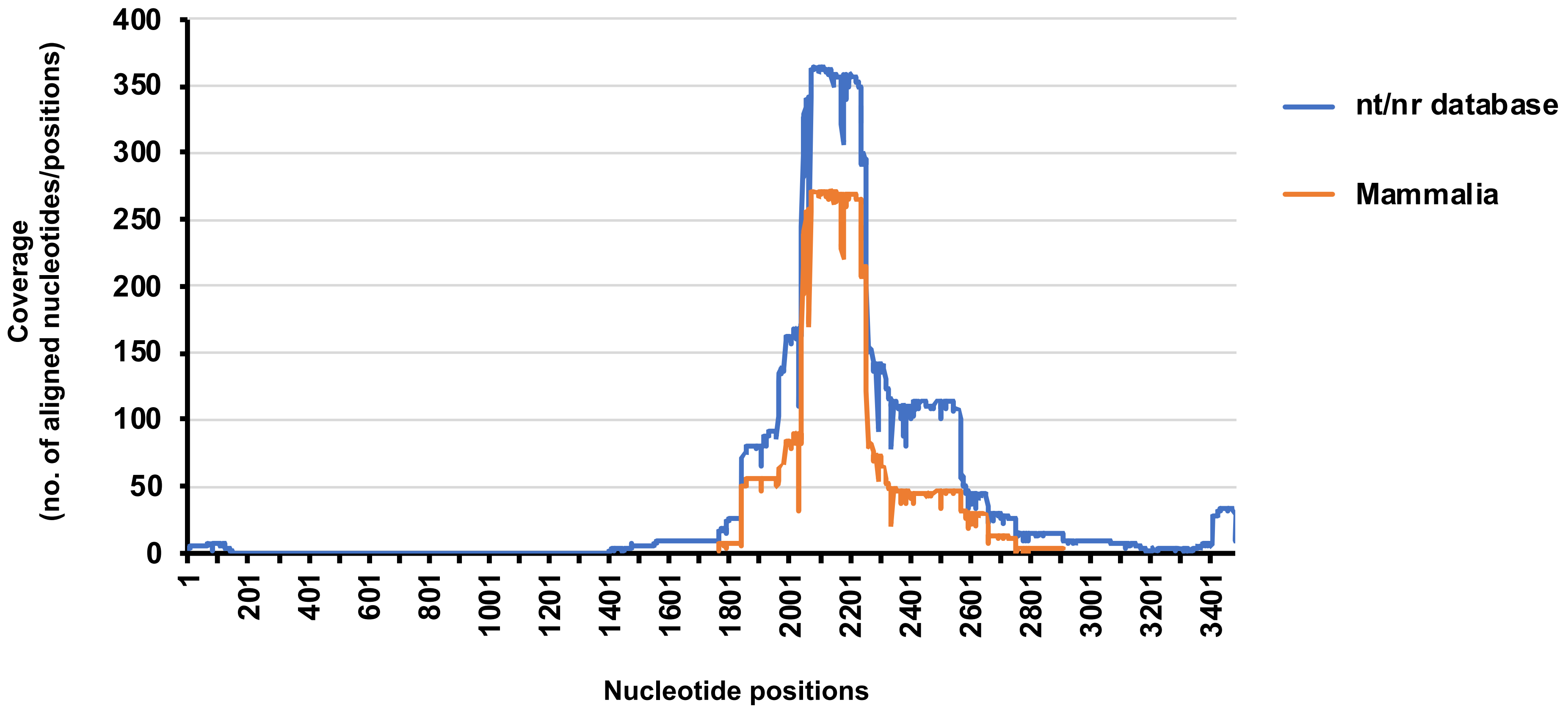

Suppl. Figure S8. Mariner-2_CPB elements coverage from BLAST significant alignments against the Genbank nucleotide database (nt/nr; blue line) and limited to “Mammalia” (orange line).

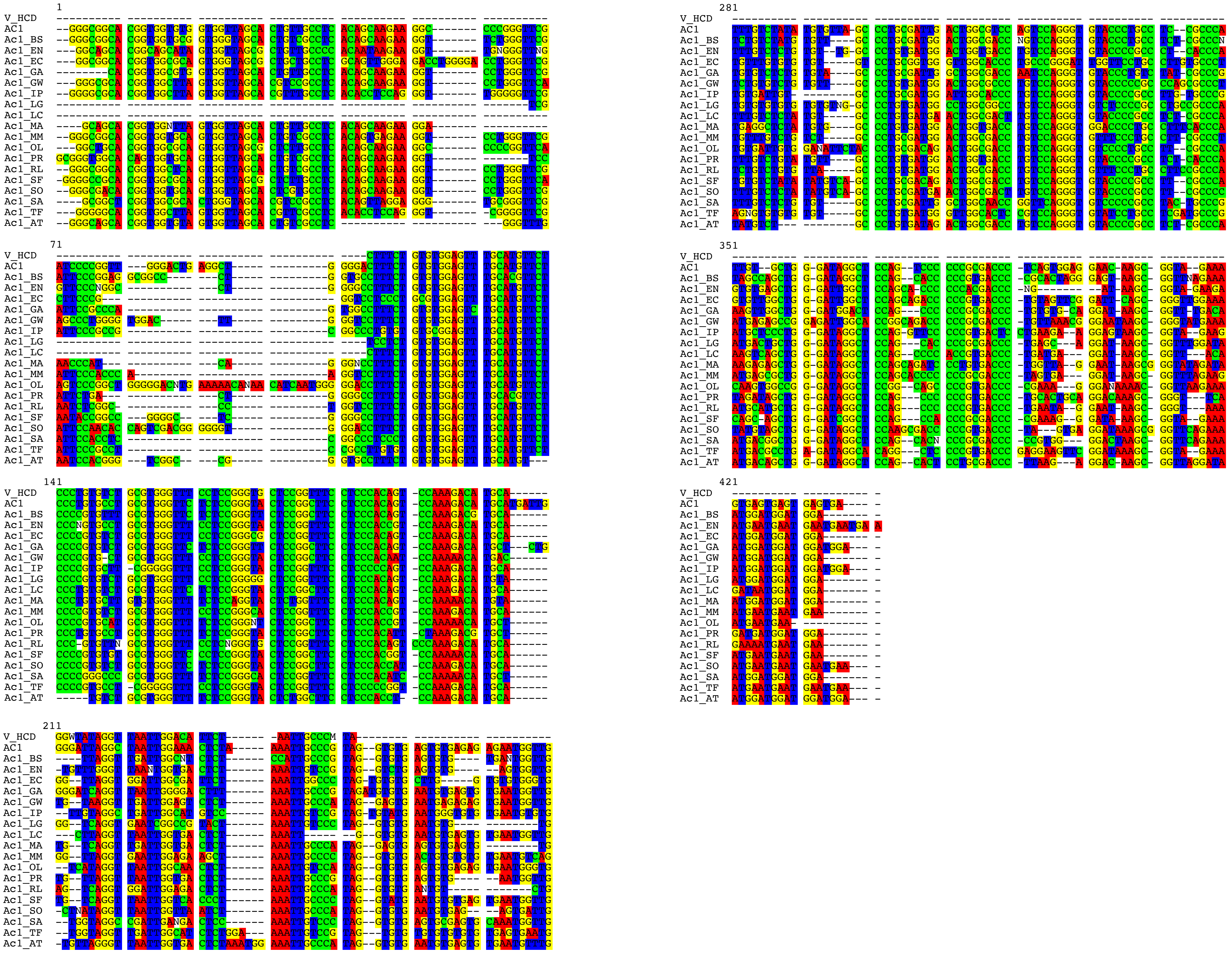

Suppl. Figure S9. Alignment of newly isolated Ac1 short interspersed elements from bony fish genomes. The original Ac1 element and the consensus sequence of V highly conserved domain are also reported. The terminal two-letter code in sequence names are the initials of the relative species, as listed in Figure 4.

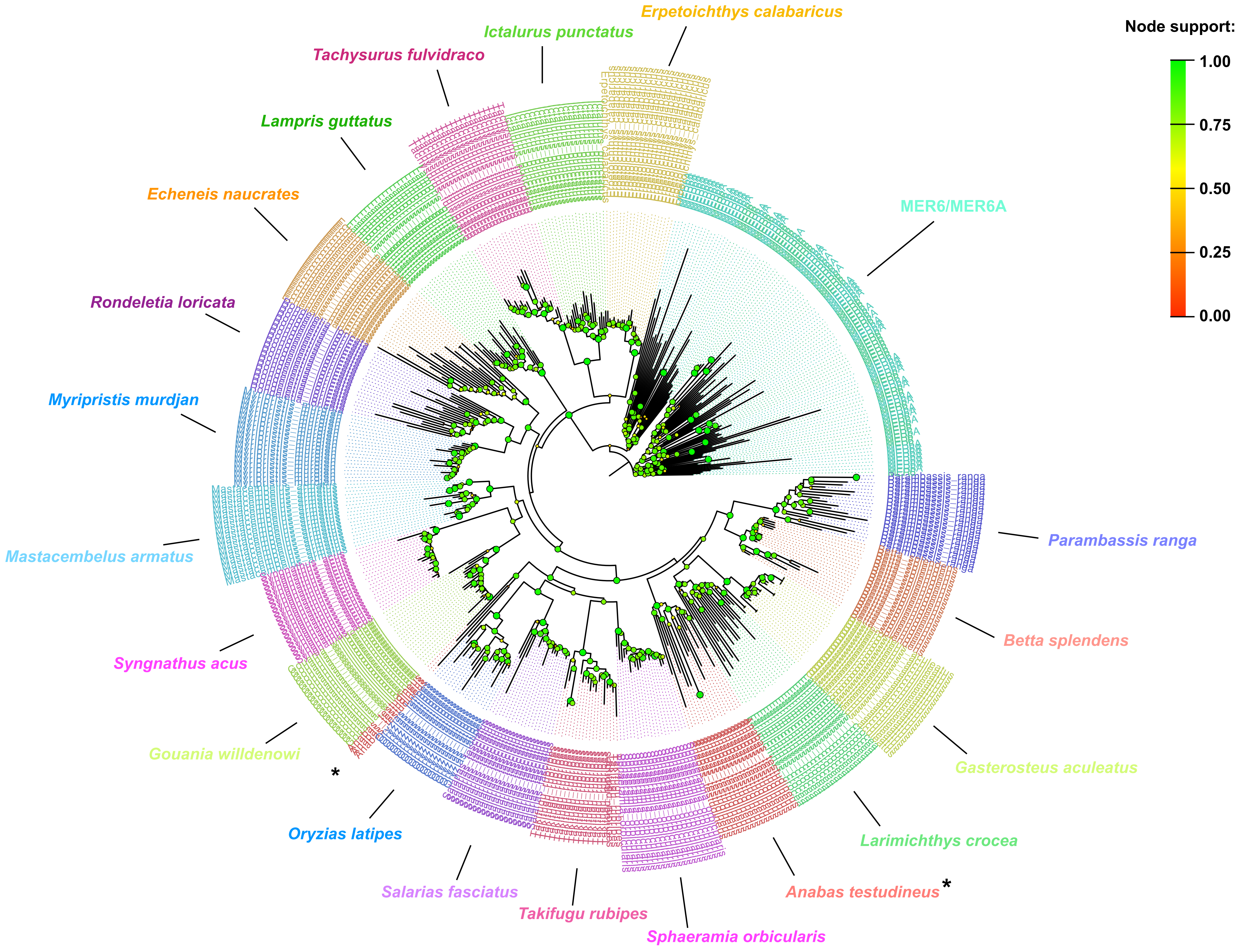

Suppl. Figure S10. Maximum likelihood tree calculated on 477 bony fish V-SINE sequences and MER6 elements, using the GTR+CAT model. Coloured dots at nodes indicate the support value, as per upper-right legend. The name of fish species from which V-SINE have been isolated are also reported near the relative cluster. Asterisks mark non-monophyletic *Anabas testudineus* Ac1-like SINEs.

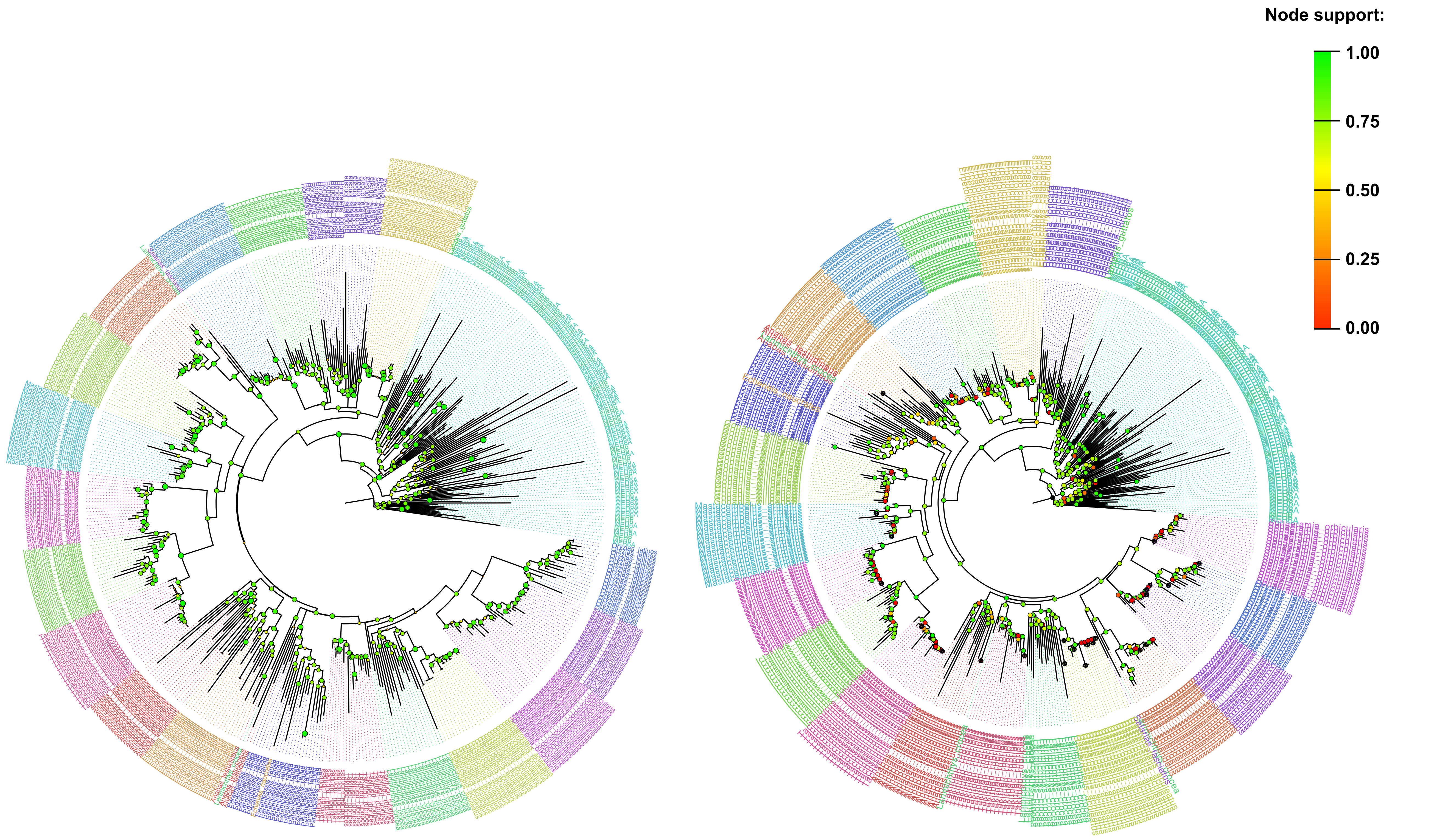

Suppl. Figure S11. Maximum likelihood tree calculated on the V HCD of 477 bony fish V-SINE sequences and MER6 elements, using the Jukes-Cantor+CAT model (left panel) or GTR+CAT (right panel). Coloured dots at nodes indicate the support value, as per upper-right legend. The name of fish species from which V-SINE have been isolated are also reported near the relative cluster. Asterisks mark non-monophyletic *Anabas testudineus* Ac1-like SINEs.

Supplementary Table S1. Jukes-Cantor divergence (%) among MER6 and MER6A repeats within each genome.

Species	MER6	MER6A
Homo sapiens	34.84	34.62
Pan troglodytes	34.92	34.69
Callithrix jaccus	41.02	39.97
Macaca mulatta	36.63	36.09
Carlito syrichta	43.21	40.19
Microcebus murinus	36.51	35.76
Pteropus vampyrus		32.32
Myotis lucifugus		35.25
Condylura cristata		56.62
Erinaceus europaeus		65.64
Solenodon paradoxus		49.96

Supplementary Table S2. Scaffold-level assembly statistics for genomes used for checking the presence of MER6/MER6A within genes.

Species	N	N50	Total Length
Homo sapiens	472	67,794,873	3,099,706,404
Pan troglodytes	4,432	53,103,722	3,050,398,082
Callithrix jacchus	16,399	5,167,444	2,914,958,544
Macaca mulatta	2,979	82,346,004	2,971,331,530
Carlito syrichta	337,189	401,181	3,453,864,774
Microcebus murinus	7,678	108,171,978	2,487,409,138
Pteropus vampyrus	36,094	5,954,017	2,198,284,804
Myotis lucifugus	11,654	4,293,315	2,034,575,300
Condylura cristata	2,040	55,520,359	1,769,662,895
Erinaceus europaeus	5,803	3,264,618	2,715,720,925

Supplementary Table S3. Genomes selected for the present study, taken from NCBI Genbank.

Class	Species	Assembly
Mammalia	*Pan troglodytes*	Clint_PTRv2
	Homo sapiens	GRCh38.p13
	Macaca mulatta	Mmul_10
	Callithrix jacchus	Callithrix jacchus-3.2
	Carlito syrichta	Tarsius_syrichta-2.0.1
	Microcebus murinus	Mmur_3.0
	Microtus ochrogaster	MicOch1.0
	Mus musculus	GRCm38.p6
	Rattus norvegicus	Rnor_6.0
	Oryctolagus cuniculus	OryCun2.0
	Sus scrofa	Sscrofa11.1
	Tupaia belangeri chinensis	TupChi_1.0
	Tursiops truncatus	Tur_tru v1
	Megaptera novaeangliae	megNov1
	Ictidomys tridecemlineatus	SpeTri2.0
	Ovis aries	Oar_rambouillet_v1.0
	Leptonychotes weddellii	LepWed1.0
	Manis tricuspis	ManTri_v1_BIUU
	Suricata suricatta	SurSur_v1_BIUU
	Tapirus indicus	TapInd_v1_BIUU
	Dicerorhinus sumatrensis	ASM284483v1
	Bos taurus	ARS-UCD1.2
	Equus caballus	EquCab3.0
	Ursus arctos horribilis	ASM358476v1
	Felis catus	Felis_catus_9.0
	Pteropus vampyrus	Pvam_2.0
	Myotis lucifugus	Myoluc2.0
	Canis lupus	ASM544666v1
	Cervus elaphus	CerEla1.0
	Erinaceus europaeus	EriEur2.0
	Condylura cristata	ConCri1.0
	Solenodon paradoxus	SolPar_v1_BIUU
	Procavia capensis	ProCapCap_v2_BIUU_UCD
	Loxodonta africana	Loxafr3.0
	Chrysochloris asiatica	ChrAsi1.0
	Orycteropus afer	OryAfe_v1_BIUU
	Dasypus novemcinctus	Dasnov3.0
	Macropus eugenii	Meug_1.1
	Sarcophilus harrisii	Devil_ref v7.0
	Monodelphis domestica	ASM229v1
	Phascolarctos cinereus	phaCin_unsw_v4.1

	Vombatus ursinus	bare-nosed wombat genome assembly
	Ornithorhynchus anatinus	mOrnAna1.p.v1
Reptilia	*Gekko japonicus*	Gekko_japonicus_V1.1
	Anolis carolinensis	AnoCar2.0
	Crotalus viridis viridis	UTA_CroVir_3.0
	Python bivittatus	Python_molurus_bivittatus-5.0.2
	Vipera berus berus	Vber.be_1.0
	Sphenodon punctatus	ASM311381v1
	Lacerta viridis	ASM90024590v1
	Pogona vitticeps	pvi1.1
	Gavialis gangeticus	ggan_v0.2
	Alligator sinensis	ASM45574v1
	Pelodiscus sinensis	PelSin_1.0
	Chrysemys picta bellii	Chrysemys_picta_BioNano-3.0.4
Aves	*Aquila chrysaetos canadensis*	Aquila_chrysaetos-1.0.2
	Chaetura pelagica	ChaPel_1.0
	Calypte anna	ASM69908v1
	Antrostomus carolinensis	ASM70074v1
	Calidris pugnax	ASM143184v1
	Charadrius vociferus	ASM70802v2
	Ciconia boyciana	Ciconia boyciana_ver1.0
	Columba livia	Cliv_2.1
	Leptosomus discolor	ASM69178v1
	Cuculus canorus	ASM70932v1
	Falco peregrinus	F_peregrinus_v1.0
	Grus japonensis	Grus japonensis_ver1.0
	Passer domesticus	Passer_domesticus-1.0
	Nestor notabilis	ASM69687v1
	Pygoscelis adeliae	ASM69910v1
	Anas platyrhynchos	IASCAAS_PekingDuck_PBH1.5
	Anser cygnoides domesticus	AnsCyg_PRJNA183603_v1.0
	Gallus gallus	GRCg6a
	Taeniopygia guttata	bTaeGut1_v1.p
	Casuarius casuarius	casCas1
	Struthio camelus australis	ASM69896v1
Amphibia	*Microcaecilia unicolor*	aMicUni1.1
	Rhinatrema bivittatum	aRhiBiv1.1
	Pyxicephalus adspersus	Pads_1.0
	Rhinella marina	RM170330
	Rana catesbeiana	RCv2.1

Nanorana parkeri	ASM93562v1
Xenopus laevis	Xenopus_laevis_v2
Ambystoma mexicanum	ASM291563v2

Suppl. Data S1. Consensus sequences of V-SINEs from Anura (frog and toads).

```
>Napa (Nanorana parkeri)
GTGGGCAGCACAGTGGCGTATGGTTAGCACTTCTGCCTTGCAGCACTGGGGGTCCCTGGTTCGAATCCCGGCCAGGACAC
TATCTGCAAGGAGTTTGCATGTTCTCCCTGTGCTTGCGTGGGTTTCCTCCGGGTACTCCGGTTTCCTCCCACACTCCAAA
GACATGCTGGTAGGTTAATTGGCTCCTGTCTAAATTGGCCCTAGTATGTGTGTGTGTGTATGTGTATGTATGCATGTGAG
ATAGGGACCTTAGATTGTAAGCTCCTTGAGGGCAGGGACTGATGTGAATGTGCAATGTATATGTAAAGCGCTGCGTAAAT
TGTTGGCGCTATATAAATACCTGTAATAAATAAA
>Pyad (Pyxicephalus adspersus)
CAGGGCAGCACGGTGGCTCAGTGGTTAGCACTCTGGCCTTTGCAGCGCTAGGTCCCAGGTTCGAATCTCAGCCAGGACAC
TATCTGCATGGAGTTTGCAGGTTCTCCCCGTGTTTGCGTGGGTTTCCTCCGGGTACTCCGGTTTCCTCCCACATTCCAAA
AACATGCAGTTAGGTTAATTGGCTTCCCCCTAAAAATTGACCTTAGACTGTATTAATGACATATGACTATGGTAGGGACA
TTAGATTGTGAGCCCCTTTGAGGGACAGCTAGTGACATGACTATGGACTTTGTACAGCGCTGCGTAATATGTTGGCGCTA
TATAAATACTGTTTAATAATAATAATAATAA
>Racat (Rana catesbeiana)
GTGGGCGGCACAGTGGTGTATGGATAGCACTTTCGCCTAGCAGTAAGAAGGGTCGCTGGTTCGAATCCCAACCACGACAC
TACCTGCCTGGAGTTTGCATGTTCTCCCTGTGCCTGCGTGGGTTTCCTCCGGGTACTCCGGTTTCCTCCCACACTCCAAA
GACATGCTGGTAGGTTAATTGGATCCTGTCTAAATTGTCCCTAATATGTGTGTGTGTGTATGTGTATGTATGAATGTGAG
TTAGGGACCTTAGATTGTAAGCTCCTTGAGGGTAGGGACTGATGTGAATGTACAATGTATATGTAAAGCGCTGCGTAAAT
TGACGGCGCTATATAAGTACCTGAAATAAATAAA
>Rhima (Rhinella marina)
CAGGGCAGCACGGTGGCTCAGTGGTTAGCACTGGTGCCTTGCAGCGCTGGGGTCCTGGGTTTGAATCCGACCAAGGACAA
CATCTGCATGGAGTTTGTATGTTCTCCCTGTGTTTGCGTGGGTTTCCTCCGGGTACTCCGGTTTCCTCCCACACTCCAAA
GACATACTGATAGGGAACTTAGATTGTGAGCCCCATTGGGGACAGTTTGATGCTAATGTCTGTAAAAGCGCTGCGGAATA
TAGTAGCGCTATATAAGTGCATAAAAATAAATAAA
```

Suppl. Data S2. Consensus sequences of V-SINEs Ac1-like from bony fishes.

```
>Ac1_BS (Betta splendens)
GGGCGGCACGGTGGTGCGGTGGGTAGCACTGTCGCCTCACAGCAAGAAGGTTCTGGGTTCGATTCCCGGAGGCGGCCCTG
GTGCCTTTCTGTGTGGAGTTTGCACGTTCTCCCCGTGTTTGCGTGGGTTCTCTCCGGGTTCTCCGGCTTCCTCCCACAGT
CCAAAGACGTGCATTAGGTTGATTGGCNTCTCTCCATTGCCCGTAGGTGTGAGTGTGTGANTGGTTGTCTGTCTATGTGT
TGCCCTGCGATGGACTGGCGACCNGTCCAGGGTGTACCCTGCCTCTCGCCCNTAGCCAGCTGGGATAGGCTCCAGCACCC
CCGTGACCCCGCACTAGGGAGTAAGCGGTTNAGAAAATGGATGGATGGA
>Ac1_EN (Echeneis naucrates)
GGCAGCACGGCAGCATAGTGGTTAGCGCTGTTGCCCCACAATAAGAAGGTTGNGGGTTNGGTTCCCNGGCCTGGGGCCTT
TCTGTGTGGAGTTTGCATGTTCTCCCNGTGCCTGCGTGGGTTTCCTCCGGGTACTCCGGTTTCCTCCCACCGTCCAAAGA
CATGCATGTTTGGGTTAANTGGTGACTCTAAATTGTCCGTAGGTCTGAGTGTGAGTGGTTGTTTGTCTCTGTGTTGGCCC
TGTGATGGACTGGTGACCTGTCCAGGGTGTACCCCGCCCTCACCCAGTGTGAGCTGGGATTGGCTCCAGCACCCCCCACG
ACCCNGATAAGCGGTAGAAGAATGAATGAATGAATGAATGAA
>Ac1_EC (Erpetoichthys calabaricus)
GGCGGCACGGTGGCGCAGTGGGTAGCGCTGCTGCCTCGCAGTTGGGAGACCTGGGGACCTGGGTTCGCTTCCCGGGTCCT
CCCTGCGTGGAGTTTGCATGTTCTCCCCGTGTCTGCGTGGGTTTCCTCCGGGCGCTCCGGTTTCCTCCCACAGTCCAAAG
ACATGCAGGTTAGGTGGATTGGCGATTCTAAATTGGCCCTAGTGTGTGCTTGGTGTGTGGGTGTGTTTGTGTGTGTGTCC
TGCGGTGGGTTGGCACCCTGCCCGGGATTGGTTCCTGCCTTGTGCCCTGTGTTGGCTGGGATTGGCTCCAGCAGACCCCC
GTGACCCTGTAGTTCGGATTCAGCGGGTTGGAAAATGGATGGATGGA
>Ac1_GA (Gasterosteus aculeatus)
CACGGTGGCGTGGTGGTTAGCACTGTTGCCTCACAGCAAGAAGGTCCTGGGTTCGATTCCGCCCAGTGGCCTTTCTGTGT
GGAGTCTGCATGTTCTCCCCGTGTCTGCGTGGGTTCTCTCCGGGTTCTCCGGCTTCCTCCCACAGTCCAAAGACATGCTC
TGGGGATCAGGTTAATTGGGGACTTTAAATTGCCCGTAGATGTGTGAATGTGAGTGTGAATGGTTGTGTGTCTCTGTGTA
GCCCTGCGATTGGCTGGCGACCAATCCAGGGTGTACCCTGTCTATCGCCCGAAGTTGGCTGGGATGGACTCCAGCCCCCC
GCGACCCTGTGTGCAGGATAAGCGGTTTGACAATGGATGGATGGATGGA
>Ac1_GW (Gouania willdenowi)
GGGCGCACGGTGGCTTAGTGGTTAGCACGTCCGCCTCACAGCAAGAAGGTCCTGGGTTCAAGCCCTGGGGTGGACTTGGG
TCCTTTCTGTGTGGAGTTTGCATGTTCTCCCCGTGCTGCGTGGGTTTCCTCCGGGTACTCCGGCTTCCTCCCACAATCCA
AAAACATGACTGTAAGGTTGATTGGAGTCTCTAAATTGCCCATAGGAGTGAATGAGAGAGTGAATGGTTGTCTGTGTCTG
TGTTGCCCTGTGATGGACTGGCGCCCTGTCCAGGGTGTACCCCCGCCCAGCGCCCTATGAGAGCCGGAGATTGGCACCGG
CAGACCCCCGCGACCCTGTTAAACGGGAATAAGCGGGTATGAAAATGGATGGATGGA
>Ac1_IP (Ictalurus punctatus)
GGGGCGCACGGTGGCTTAGTGGTTAGCACGTTTGCCTCACACCTCCAGGGTTGGGGGTTCGATTCCCGCCGCGGCCCTGT
GTGTGCGGAGTTTGCATGTTCTCCCCGTGCTTCGGGGGTTTCCTCCGGGTACTCCGGTTTCCTCCCCCAGTCCAAAGACA
```

TGCATTGTAGGCTGATTGGCATGTCCAAATTGTCCGTAGTGTATGAATGGGTGTGTGAATGTGTGTGTGATTGTGCCCTG
CGATGGATTGGCACCCTGTCCAGGGTGTACCCCGCCTTGTGCCCGATGCTCCCTGGGATAGGCTCCAGGTTCCCCCGTGA
CTCCTGAAGAAGGAGTAAGCGGTAGAAGATGGATGGATGGATGGA
>Ac1_LG (*Lampris guttatus*)
TCGTCCTCTGTGTGGAGTTTGCATGTTCTCCCCGTGTCTGCGTGGGTTTCCTCCGGGGGCTCCGGTTTCCTCCCACAGTC
CAAAGACATGTAGGTCAGGTGAATCGGCCGTACTAAATTGTCCCTAGGTGTGAATGTGTGTGTGTGTGTGTGTGTNGGCC
CTGTGATGGCCTGGCGGCCTGTCCAGGGTGTCTCCCCGCCTGCCGCCCAATGACTGCTGGGATAGGCTCCAGCACCCCGC
GACCCTGAGCAGGATAAGCGGTTTGGATAATGGATGGATGGA
>Ac1_LC (*Larimichthys crocea*)
CTTTCTGTGTGGAGTTTGCATGTTCTCCCTGTGTCTGCGTGGGTTCTCTCCGGGTACTCCGGCTTCCTCCCACAGTCCAA
AGACATGCACTTAGGTTAATTGGTGACTCTAAATTGGTGTGAATGTGAGTGTGAATGGTTGTTTGTCTCTATGTGCCCTG
TGATGAACTGGCGACTTGTCCAGGGTGTACCCCGCCTCTCGCCCAAAGTCAGCTGGGATAGGCTCCAGCCCCACCGTGAC
CCTGATGAGGATAAGCGGTTACAGATAATGGATGGA
>Ac1_MA (*Mastacembelus armatus*)
GCAGCACGGTGGNTTAGTGGTTAGCACTGTTGCCTCACAGCAAGAAGGAAACCCATCAGGGNCCTTTCTGTGTGGAGTTT
GCATGTTCTCCCTGTGCTTGTGTGGGTTTTCTCCAGGTACTCTGGTTTCCTCCCACAGTCCAAAAACATGTATGTCAGGT
TGATTGGTGACTCTAAATTGCCCATAGGAGTGAGTGTGAGTGTGTGAGGCTCTATGTGGCCCTGTGATGGACTGGTGACC
TGTCCAGGGTGGACCCCTGCCTTTCACCCAAAGAGAGCTGGGATAGGCTCCAGCAGATCCCTGTGACCCTGGTTAGGAAT
AAGCGGGTATAGATAATGGATGGATGGA
>Ac1_MM (*Myripristis murdjan*)
GGGCGGCACGGTGGTGCAGTGGTTAGCACTGTCGCCTCACAGTGAGAAGGTCCTGGGTTCGATTCCCACCCAAGGTCCTT
TCTGTGTGGAGTTTGCATGTTCTCCCCGTGTCTGCGTGGGTTTCCTCCGGGCACTCCGGTTTCCTCCCACCGTCCAAAGA
CATGCAGGTTAGGTGAATTGGAGAAGCTAAATTGCCCCTAGGTGTGACTGTGTGTGTGAATGTCAGTGTTTGTCTGTCTG
CCCTGCGATGGACTGGCGACCTGTCCAGGGTGTTTCCCTGCCTTCGCCCTATGAGCGCTGGGATAGGCTCCAGCACCCCC
CCGCGACCCTAGTGAGGATAAGCGGTTTAGAAGATGAATGAATGAA
>Ac1_OL (*Oryzias latipes*)
GGCTGCACGGTGGCGCAGTGGTTAGCGCTCTTGCCTCACAGCAAGAAGGCCCCCGGTTCAAGTCCCGGCTGGGGGACNTG
AAAAACANAACATCAATGGGGGACCTTTCTGTGTGGAGTTTGCATGTTCTCCCCGTGCATGCGTGGGTTTTCTCCGGGNT
CTCCGGCTTCCTCCCACCGTCCAAAAACATGCTTCATAGGTTAATTGGCAACTCTAAATTGTCCATAGGTGTGAGTGTGA
GAGTGAATGGGTGTGTGATTGTGGANATTCTACCCTGCGACAGACTGGCGACCTGTCCAGGGTGTCCCCTGCCTTCGCCC
ACAAGTGGCCGGGATAGGCTCCGGCAGCCCCGTGACCCCGAAAGGGANAAAACGGTTAAGAAAATGAATGAA
>Ac1_PR (*Parambassis ranga*)
GCGGGTGGCACAGTGGTGCAGTGGTTAGCACTGTCGCCTCACAGCAAGAAGGTTCCATTCTGACTGGGGCCTTTCTGTGT
GGAGTTTGCACGTTCTCCCTGTGCCTGCGTGGGTTTTCTCCGGGTACTCCGGCTTCCTCCCACATTCTAAAGACGTGCTT
GTTAGGTTAATTGGTGACTCTAAATTGCCCGTAGGTGTGAGTGTGAATGGTTGTTTGTCTGTATGTTGCCCTGTGATGGA
CTGGTGACCTGTCCAGGGTGTACCCCGCCTCTCACCCATAGATAGCTGGGATAGGCTCCAGCCCCCCGTGACCCTGCACT
GCAGGACAAAGCGGGTTCAGATGATGGATGGA
>Ac1_RL (*Rondeletia loricata*)
GGGCGGCACGGTGGCTCAGTGGTTAGCACTGTCGCCTCACAGCAAGAAGGTCCTGGGTTCGAATCTCGGCCCTGGTCCTT
TCTGTGTGGAGTTTGCATGTTCTCCCGTGTTNGCGTGGGTTTCCTCNGGGTGCTCCGGTTTCCTCCCACAGTCCCAAAGA
CATGCAAGTCAGGTGGATTGGAGACTCTAAATTGCCCATAGGTGTGANTGTCTGTCTGTCTGTGTTAGCCCTGTGATGGA
CTGGCGACCTGTCCAGGGTGTTTCCCTGCCTTCCGCCCAATGCATGCTGGGATAGGCTCCAGCCCCCCGTGACCCTGAAT
AGGAATAAGCGGGTAAAGAAAATGAATGAA
>Ac1_SF (*Salarias fasciatus*)
GGGGCCGCACGGTGGCGCAGTGGTTAGCGCTCTTGCCTCACAGCAAGAAGGTCCTGGGTTCAAATACCGGCCGGGGCTCG
GGGCCTTTCTGTGTGGAGTTTGCATGTTCTCCCCGTGTGTGCGTGGGTTCCCTCCGGGTACTCCGGCTTCCTCCCACGGT
CCAAAAACATGCATGTCAGGTTAATTGGTCACCCTAAATTGCCCCTAGGTATGAATGTGTGAGTGAATGGTTGTGTGTCT
ATATATGTCAGCCCTGCGACAGACTGGCGACCTGTCCAGGGTGTACCCCGCCTTCGCCCACAGCAGCTGGGATCGGCTCC
AGCCACCCGCGACCCCGAAAGGGATAAAGCGGTAGAAAATGAATGAATGAA
>Ac1_SO (*Sphaeramia orbicularis*)
GGGCGACACGGTGGTGCAGTGGTTAGCACTCGTGCCTCACAGCAAGAAGGTCCTGGGTTCGATTCCAACACCAGTCGACG
GGGGGTGGGACCTTTCTGTGTGGAGTTTGCATGTTCTCCCCGTGTCTGCGTGGGTTCTCTCCGGGTACTCCGGCTTCCTC
CCACCATCCAAAGACATGCACTNATAGGTTAATTGGTTAATCTAAATTGCCCATAGGTGTGAATGTGAGAGTGATTGTTT
GTCTCTATATGTCAGCCCTGCGATGAACTGGCGACTTGTCCAGGGTGTACCCCGCCTTCGCCCCTATGTAGCTGGGATAG
GCTCCAAGCGACCCCCGTGACCCTAGTGAGGATAAAGCGGGTTCAGAAAATGAATGAATGAATGAA
>Ac1_SA (*Syngnathus acus*)
GCGGCTCGGTGGCGCACTGGGTAGCACGTCCGCCTCACAGTTAGGAGGGTGCGGGTTCGATTCCACCTCCGGCCCTCCCT
GTGTGGAGTTTGCATGTTCTCCCCGGGCCCGCGTGGGTTTTCTCCGGGCACTCCGGTTTCCTCCCACATCCCAAAAACAT
GCTTGGTAGGCCGATTGANGACTCCAAATTGTCCCTAGGTGTGAGTGCGAGTGCAAATGGTTGTTTGTCTCTGTGTGCCC
TGCGATTGGCTGGCAACCGGTTCAGGGTGTCCCCCGCCTACTGCCCGATGACGGCTGGGATAGGCTCCAGCACNCCCGCG
ACCCCCGTGGGGACTAAGCGGTTCAGAAAATGGATGGATGGA

>Ac1_TF (*Tachysurus fulvidraco*)
GGGGGCACGGTGGCTTAGTGGTTAGCACGTTCGCCTCACACCTCCAGGGTCGGGGTTCGATTCCCGCCTCCGCCTTGTGT
GTGTGGAGTTTGCATGTTCTCCCCGTGCCTCGGGGGTTTCCTCCGGGTACTCCGGTTTCCTCCCCCGGTCCAAAGACATG
CATGGTAGGTTGATTGGCATCTCTGGAAAATTGTCCGTAGTGTGTGTGTGTGTGAGTGAATGAGNGTGTGTGTGTGCC
CTGTGATGGGTTGGCACTCCGTCCAGGGTGTATCCTGCCTCGATGCCCGATGACGCCTGAGATAGGCACAGGCTCCCCGT
GACCCGAGGAAGTTCGGATAAAGCGGTAGAAAATGAATGAATGAATGAA
>Ac1_AT (*Anabas testudineus*)
GGGCAGCACGGTGGTGTAGTGGTTAGCACTGTCGCCTCGGGTTTGAATCCACGGGTCGGCCGGGTGCCTTTCTGTGTGGA
GTTTGCATGTTGTCTGCGTGGGTTTTCTCCGGGTACTCTGGCTTCCTCCCACCTCCAAAGACATGCATGTTAGGGTTAAT
TGGTGACTCTAAATGGAAATTGCCCATAGGTGTGAATGTGAGTGTGAATGTTTGTATGTCTGCCCTGTGATAGACTGGCG
ACCTGTCCAGGGTGTACCCCGCCTCTCGCCCAATGACAGCTGGGATAGGCTCCAGCACTCCTGCGACCCTTAAGAGGACA
AGCGGTTAGGATAATGGATGGATGGATGGA
